# Clinical Evidence and Potential Mechanisms of Complementary Treatment of *Ling Gui Zhu Gan* Formula for the Management of Serum Lipids and Obesity

**DOI:** 10.1155/2022/7714034

**Published:** 2022-05-09

**Authors:** Jiashuai Huang, Linjing Zhao, Jijia Sun, Lixin Wang, Jianrong Gu, Xijian Liu, Mengwen Yang, Yuting Wang, Ning Zhang, Jiamin Zhu, Shanshan Xu, Xinfeng Ren, Ying Su

**Affiliations:** ^1^College of Chemistry and Chemical Engineering, Shanghai University of Engineering Science, Shanghai 201620, China; ^2^Department of Mathematics and Physics, Pharmacy School, Shanghai University of Traditional Chinese Medicine, Shanghai 201203, China; ^3^Integrated TCM & Western Medicine Department, Shanghai Pulmonary Hospital, Tongji University, Shanghai 200433, China; ^4^Informatization Office, Shanghai University of Engineering Science, Shanghai 201620, China; ^5^School of Basic Medical Sciences, Chengdu University of Traditional Chinese Medicine, Chengdu 611137, Sichuan Province, China; ^6^Department of Pathology, School of Basic Medical Sciences, Fudan University, Shanghai 200032, China

## Abstract

**Objective:**

This study aims to evaluate the clinical effects of *Ling Gui Zhu Gan* formula (LGZG), a famous TCM formula, for the management of serum lipids and obesity and preliminarily elucidates the bioactive components and the potential mechanism.

**Methods:**

Cluster analysis was adopted to investigate the TCM herbs and their frequency of occurrence for treating hyperlipidemia and obesity in an academic experience database of Chinese famous TCM doctors (http://www.gjmlzy.com:83). Then, relevant randomized controlled trials (RCTs) about LGZG supplementation in improving lipid levels and obesity were retrieved and analyzed. Lastly, the integration of network pharmacology, as well as greedy algorithms, which are theoretically well founded for the set cover in computer science, was exploited to identify the bioactive components of LGZG and to reveal potential mechanisms for attenuation or reversal of hyperlipidemia and obesity.

**Results:**

Based on the cluster analysis of 104 cases in TCM academic experience database, four TCM herbs in LGZG showed high-use frequency for treating hyperlipidemia and obesity. Meta-analysis on 19 randomized controlled trials (RCTs) with 1716 participants indicated that LGZG supplementation significantly decreased the serum levels of total triglycerides, total cholesterol, low-density lipoprotein cholesterol, BMI, and body weight and increased high-density lipoprotein cholesterol, compared with clinical control groups. No serious adverse effect was detected in all studies. Twenty-one bioactive components of LGZG, mainly flavonoids (i.e., naringenin, kaempferol, and kumatakenin), saponins (i.e., hederagenin), and fatty acids (i.e., eicosenoic acid), had the potential benefits possibly by regulating multiple targets such as PTPN1, CYP19A1, and ESR2, as well as a few complex pathways including the TNF signaling pathway, PPAR signaling pathway, arachidonic acid metabolism, fat digestion, and absorption.

**Conclusion:**

The present study has proved the clinical value of LGZG as a complementary treatment for attenuation or reversal of hyperlipidemia and obesity. More high-quality clinical and experimental studies in the future are demanded to verify its effects and the precise mechanism of action.

## 1. Introduction

Dyslipidemia is a worldwide prevalence health hazard which acts as a major risk factor for coronary artery disease and stroke [1, 2]. Also, increasing evidences have emphasized the decisive role of lipid metabolic disturbance in tumor proliferation and metastasis [3]. The typical characteristic of dyslipidemia included the elevation of serum total triglycerides (TG), cholesterol (TC), and low-density lipoprotein cholesterol (LDL-c) and relative reduction of high-density lipoprotein cholesterol (HDL-c). The front-line therapy for the treatment of high serum lipid levels is statin medication, which significantly reduced the risk of cardiovascular events and cardiovascular mortality [4, 5]. Unfortunately, statins can have undesirable adverse effects such as myopathy, transaminase elevations, and an increased risk of incident diabetes mellitus among some patients, which can hinder medication compliance [6]. Accumulating evidence has indicated that obesity is closely related to an increased risk of dyslipidemia and other metabolic disorders and taking synthetic antiobesity medications exerts some adverse effects and often its efficacy is attenuated after prolonged use [7]. Therefore, new treatments are needed for the management of dyslipidemia and obesity.

**Figure 1:**
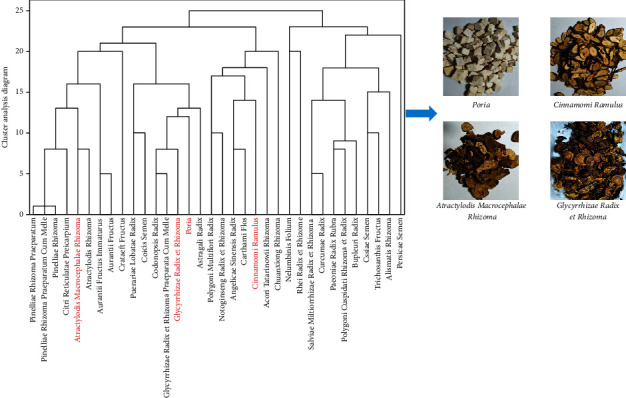
Cluster analysis of 104 cases associated with hyperlipidemia/dyslipidemia and obesity using an academic experiences database of Chinese famous TCM doctors (http://www.gjmlzy.com:83) and the herbs composition of LGZG.

TCM herbal formulae have been proven safe and effective as a complementary and alternative medical treatment for various chronic diseases [8], even for the ongoing outbreak of coronavirus disease 2019 (COVID-19) [9]. Increasing evidence shows that certain classic TCM formulae are clinically reliable for the improvement of hyperlipidemia and obesity. Hence, we collected 104 cases reported in the National Service Platform for Academic Experience of Famous TCM doctor (http://www.gjmlzy.com:83) or in China National Knowledge Internet database (http://www.cnki.net). Through the data mining of pesticide effects, flavor, property, and meridian tropism and cluster analysis, a total of 34 TCM herbs with a use frequency of more than eight were obtained and listed in Table S1. It has been found that a well-known TCM herbal formula of Ling Gui Zhu Gan (LGZG), which consists of *Poria* (Fu Ling, *Poria cocos (Schw.) Wolf*), *Cinnamomi ramulus* (Gui Zhi, *Cinnamomum cassia Presl*), *Atractylodis macrocephalae Rhizoma* (Bai Zhu, *Atractylodes macrocephala Koidz.*), and *Glycyrrhizae radix et rhizoma* (Gan Cao, *Glycyrrhiza uralensis Fisch.*) at the ratio of 4 : 3:3 : 2, usually serves as the basic recipe for the management of serum lipids and obesity (Figure 1).

LGZG, first recorded in the *Synopsis of Prescriptions of the Golden Chamber*, has been traditionally applied for treating patients with spleen deficiency and dampness syndrome in China. Studies on the compatibility of composite herbal medicines in LGZG highlighted the theory of TCM that *Poria* and *Cinnamomi ramulus* are the basis, while *A. macrocephalae rhizoma* and *Glycyrrhizae radix et rhizoma* are the adjuvants [10]. Traditional decoction [11] and granules [12], the most common two dosage forms, are prepared by standardized methods, respectively. In recent years, a few randomized clinical trials (RCTs), which investigated the potential lipid-lowering effects of original or modified LGZG alone, or LGZG combined with routine treatment strategies such as western medicines (WM), dietary intervention and physical activity, have shown the dramatic efficacy for serum lipids control and obesity management. However, no relevant systematically evaluation has been reported, thus far.

The chemical characterization of original and modified LGZG formulations was identified, and the quality of preparation was controlled using the key effective components of glycyrrhizic acid and others such as dehydrotumulosic acid and cinnamic acid (Figure S1) under high-performance liquid chromatography [11, 13, 14]. In addition, many active ingredients found in these herbs consisting of LGZG or modified LGZG have been postulated to be effective, mainly including flavonoids, lipoid, coumarin and its glycosides, cardenolide, saponins, steroids and triterpenes, polysaccharides, tannin, phenols, organic acids, and others [15]. To the best of our knowledge, the regulatory mechanism of multicomponents and multitargets interactive network of LGZG for treatment of hyperlipidemia and obesity remains unclear, however.

Network pharmacology occurring recently can effectively elucidate the interaction between active components, targets, and disease phenotype, and therefore, plays a vital role in exploring therapeutic mechanism of TCM [16]. Greedy algorithms, as a theoretically well-founded technology for the set cover in computer science [17, 18], can also be adopted in finding the minimized set of bioactive components with satisfying cover of targets associated with drug and disease. The present study aims to systematically review the clinical efficacy of LGZG supplementation for attenuation or reversal of hyperlipidemia and obesity, as well as to reveal the bioactive components of LGZG and their potential mechanism of action, through an integrated approach of network pharmacology and greedy algorithms.

## 2. Methods

### 2.1. Data Sources and Searching Strategies

The present systematic review and meta-analysis were designed and performed based on the guidelines of the PRISMA statement (Table S2) [19].

Comprehensive information retrieval was performed by two reviewers (JH and YW) independently. The databases include PubMed (http://www.ncbi.nlm.nih.gov/pubmed), EMbase (https://www.elsevier.com/solutions/embase-biomedical-research), Cochrane Library (http://www.cochranelibrary.com/), China Scientific Journals Full-Text Database (VIP) (http://www.cqvip.com/), Wanfang Database (http://www.wanfangdata.com.cn/), and China National Knowledge Infrastructure Database (CNKI) (http://www.cnki.net/). Dates ranged from the inception to Jun. 30, 2021. Any disagreement was discussed until the final agreement was reached.

The following key terms were searched for English and Chinese databases: “lingguizhugan (Ling Gui Zhu Gan in Pinyin)” OR “LGZG (only used in the English strategy” in combined with “dyslipidemia (Xue Zhi Yi Chang in Chinese)” OR “hyperlipidemia (Gao Zhi Xue Zheng in Chinese)” OR “obesity (Fei Pang in Chinese)” OR “triglyceride (Gan You San Zhi in Chinese)” OR “total cholesterol (Zong Dan Gu Chun in Chinese)” OR “high-density lipoprotein (Gao Mi Du Zhi Dan Bai in Chinese)” OR “low-density lipoprotein (Di Mi Du Zhi Dan Bai in Chinese)” OR “BMI”. Whenever possible, Medical Subject Headings (MESH) terms were used. Besides, a snowballing method searching the bibliographies of retrieved references was applied to identify potentially relevant articles. The electronic search strategy is shown in Table S3, taking Cochrane Library as an example.

### 2.2. Study Selection

The inclusion criteria for articles were as follows: (1) The studies were randomized controlled trials in patients with dyslipidemia that meet the diagnostic criteria of 2016 Chinese guideline for the management for dyslipidemia in adults [20], with or without other metabolic disorders. (2) The experiment group was applied with original or modified LGZG alone, or LGZG combined with other treatments including western therapeutic agents such as statin or fibrate, dietary intervention, exercise, health education, and others. The control group applied a single WM treatment or nondrug therapy such as dietary intervention and exercise, health education, and others. (3) Measurement outcomes included two or more of lipid parameters of TG, TC, LDL-c, and HDL-c, with or without obesity indices such as BMI, body weight (BW) and waist circumference (WC).

The exclusion criteria for articles were as follows: (1) duplicated citations or publications; (2) obviously irrelevant studies including *in vitro* studies, animal studies, or other conditions such as surgery and radiotherapy; (3) nonrandomized controlled studies and other unqualified studies; (4) data inaccessible in some conference papers.

### 2.3. Data Extraction

Data extraction was independently performed by two researchers (JH and LZ) and disagreements were resolved by consensus. The data were recorded using an extraction sheet including the first author of the study and year of publication; sample size; average age, sex, and course of disease of the subjects; interventions in the experiment and control groups; treatment dosage and duration, and outcomes indicators; and others [19]. Serum lipid levels and obesity parameters in each study were also extracted before and after the treatment. The information about adverse reaction was also recorded.

### 2.4. Risk of Bias Assessment

The “risk of bias tool” of the Cochrane Collaboration was used to assess the risk of bias in the included RCTs by two researchers (LZ and JH). The assessment criteria include seven aspects: random sequence generation (selection bias), allocation concealment (selection bias), the blindness of participants and personnel (performance bias), the blindness of outcome assessment (detection bias), incomplete outcome data (attrition bias), selective reporting (reporting bias), and other bias. Those that meet the standard test were classified as low risk of bias, and those that do not meet the standard test were classified as high risk of bias. If the information was inadequate to form a judgment, it was classified as insufficient to make a risk judgment. In case of any disagreement, a third researcher (MY) extracted the data, and the results were attained by consensus.

The Jadad scoring scale [21] was used to evaluate the included RCTs in three aspects (1–5 points). Low-quality research was 1-2 points, and high-quality research was 3–5 points. The evaluation contents include random sequence, blind method, and withdrawal. Exactly, the study describing the random grouping method or blind method correctly was counted as 2 points, respectively, and that mentioning the “random grouping” or “double-blind” but not describing the method was counted as 1 point. And the study describing the number of withdrawals or loss of follow-up cases and explaining the reasons was worth 1 point. The measurement of the researcher agreement was done using kappa statistics [22]. Based on the kappa values, the level of agreement was defined as almost perfect (0.81–1.00), substantial (0.61–0.80), moderate (0.41–0.60), fair (0.21–0.40), slight (0.00–0.20), and poor (<0.00).

### 2.5. Statistical Analysis

Meta-analysis was performed by Cochrane Review Manager 5.3 (Copenhagen: The Nordic Cochrane Centre, The Cochrane Collaboration, 2014). Dichotomous data were expressed as risk ratio (RR) and continuous variables as the mean differences (MD) with 95% confidence intervals (95% CI). I-squared (*I*^2^) statistic was used to assess statistical heterogeneity. *I*^2^ values greater than 50% were considered indicative of high heterogeneity [23]. Data with substantial heterogeneity (*I*^*2*^ *>* 50% and *p* < 0.05) was assessed as a random-effects model, whereas others were assessed as a fixed-effects model. Sensitivity analysis and subgroup analysis were then adopted to determine the robustness of the results, when possible, by removing one study at a time. Finally, the funnel plots and Begg's linear regression test by Stata 11.0 software (StataCorp LP, College Station, TX) were used to evaluate potential publication bias, and a *p* < 0.05 was statistically significant [24, 25].

### 2.6. Evidence Quality Evaluation

The Grading of Recommendations Assessment, Development, and Evaluation (GRADE) methodology was applied to assess the certainty of evidence [26, 27], using the GRADE pro Guideline Development Tool accessible from gradepro.org. The RCT was preset to the highest level of evidence in the GRADE evidence quality assessment, and whether degradation was considered according to five domains including risk of bias, indirectness, inconsistency, imprecision, or publication bias. The grades of evidence were classified as high quality, moderate quality, low quality, and very low quality.

### 2.7. Network Pharmacology Analysis

#### 2.7.1. Chemical Component Screening

The chemical components in LGZG were collected from Traditional Chinese Medicine Systems Pharmacology Database and Analysis Platform (TCMSP, http://www.tcmspw.com) [28]. Then, compounds with oral bioavailability (OB) ≥ 30% [29] and drug-likeness (DL) ≥ 0.18 [30] were filtrated for subsequent analysis.

#### 2.7.2. Key Targets Identification

The similarity ensemble approach (SEA, http://sea.bkslab.org/) [31], SwissTargetPrediction (http://www.swisstargetprediction.ch/) [32], and STITCH (http://stitch.embl.de/) [33] databases were used to search for putative targets of active components in LGZG. For targets related to diseases, three key terms, namely hyperlipidemia, dyslipidemia, and obesity, were searched in the Therapeutic Target Database (TTD, http://db.idrblab.net/ttd/) [34], DrugBank (https://www.drugbank.ca/) [35], and DisGeNET (https://www.disgenet.org/) [36] databases. The gene names and Uniprot ID of protein targets were normalized using the Uniprot database (https://www.uniprot.org/) [37].

#### 2.7.3. Gene Ontology and KEGG Pathway Enrichment Analysis

Gene ontology (GO) and KEGG pathway enrichment analyses were carried out using the Database for Annotation, Visualization, and Integrated Discovery system (DA-VID, http://david.abcc.ncifcrf.gov/home.jsp) [38, 39] . Three GO terms, including the biological process (BP), cellular component (CC), and molecular function (MF) categories [40], as well as key KEGG pathways information, were diagramed using SangerBox software (http://sangerbox.com/Tool).

#### 2.7.4. Network Construction

Protein-protein interaction (PPI) network was acquired from the STRING database (https://string-db.org/, version 10.5) [41]. The topological features of PPI network were calculated, and key targets were identified through comparing the degree values in PPI. In addition, herb-component-target-pathway interaction network was constructed with Cytoscape 3.7.1 software (https://cytoscape.org/), an open-source software platform for visualizing complex networks.

#### 2.7.5. Greedy Algorithms for Finding a Minimized Set of Bioactive Components

The minimized set of bioactive components of LGZG which could totally cover the targets associated with drugs and diseases were obtained through the use of greedy algorithms [17, 18]. Details to explain the calculation of the greedy algorithm were provided in supplementary material.

## 3. Results

### 3.1. Study Selection

**Figure 2:**
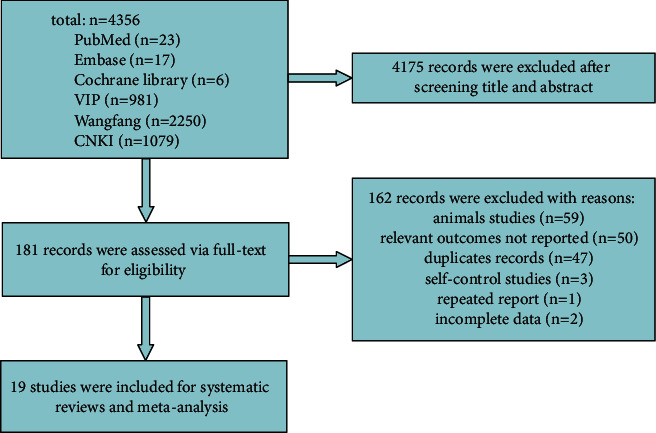
Flow diagram of records inclusions.

A total of 4356 studies were retrieved from the database, excluding the unrelated articles based on titles and abstracts, and the full text of 181 articles for further screening. Among them, 19 eligible studies [42–60] are included in the meta-analysis, and 162 were excluded with the reasons provided in Figure 2. Details of the characteristics of these included studies are summarized in Table 1. The original data regarding outcome indicators extracted from the eligible studies are shown in Table 2.

### 3.2. Risk Bias Assessment of Included Studies

**Figure 3:**
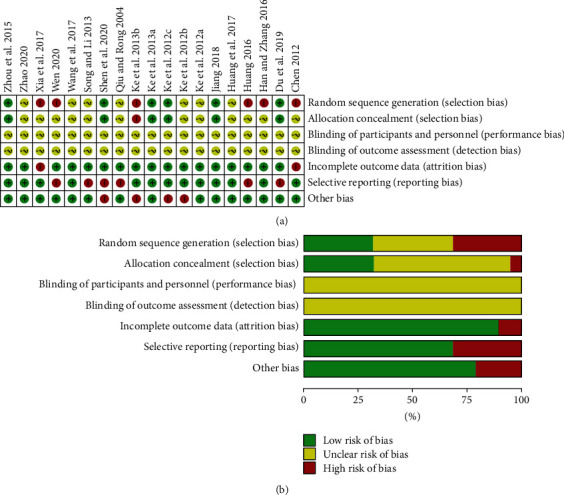
The risk of methodological bias. (a) The risk of bias summary: authors' judgments about each risk of bias item for each included study; (b) the risk of bias graph.

All studies were evaluated independently by two researchers according to the Cochrane risk of bias assessment tool, and the summary of risks of bias is presented in Figure 3. Six of the included studies [37, 38, 44, 49, 51, 53] performed random assignment by the order of visits or hospitalization, and one [44] was lack of the information about the allocation concealment. None of the studies provided the detail about the blinding of the participants, personnel, and outcome assessment. Two studies [44, 60] failed to report the BMI after the intervention, and six studies [45, 47–49, 56, 59] had the incompleteness of two primary outcome indicators of LDL-c and HDL-c, which could cause the attribution and reporting biases. Four studies [48, 51, 53, 54] were treated with short-term fasting or calorie-restricted diets in experiment group while not in controls, which were different from others. Jadad scores of the included studies by two raters were shown in Table S4. Kappa statistics showed a value of 0.883 (*p* < 0.001), highlighting an almost perfect agreement of the judgment on the quality of the included studies among two authors (Table 3).

### 3.3. Results of Meta-Analysis

#### 3.3.1. Effect of LGZG on Clinical Efficacy Rate

**Figure 4:**
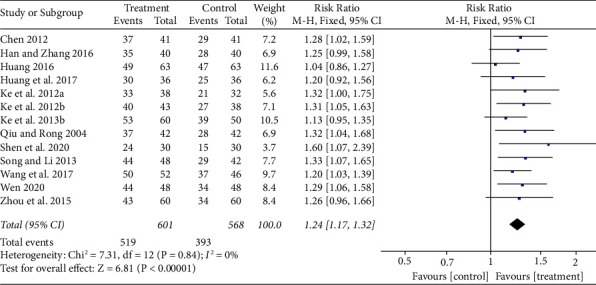
Forest plot of the effect of LGZG on the effective rate.

A total of 13 articles (68%) reported that the efficacy rate was between the experiment and control groups, of which 8 RCTs [42, 47, 49–51, 54, 56, 57]were treated with LGZG alone in the experiment group, whereas the remaining studies [45, 46, 48, 58, 60] reported a combination treatment of LGZG with WM. Results showed that the clinical effective rate of the treatment group was better than the control (RR, 1.24; 95% CI: 1.17 to 1.32; *p* < 0.00001), as shown in Figure 4. No heterogeneity was located (*I*^2^ = 0%).

#### 3.3.2. Effect of LGZG on Serum Lipids Profile of TG, TC, LDL-C, and HDL-C

**Figure 5:**
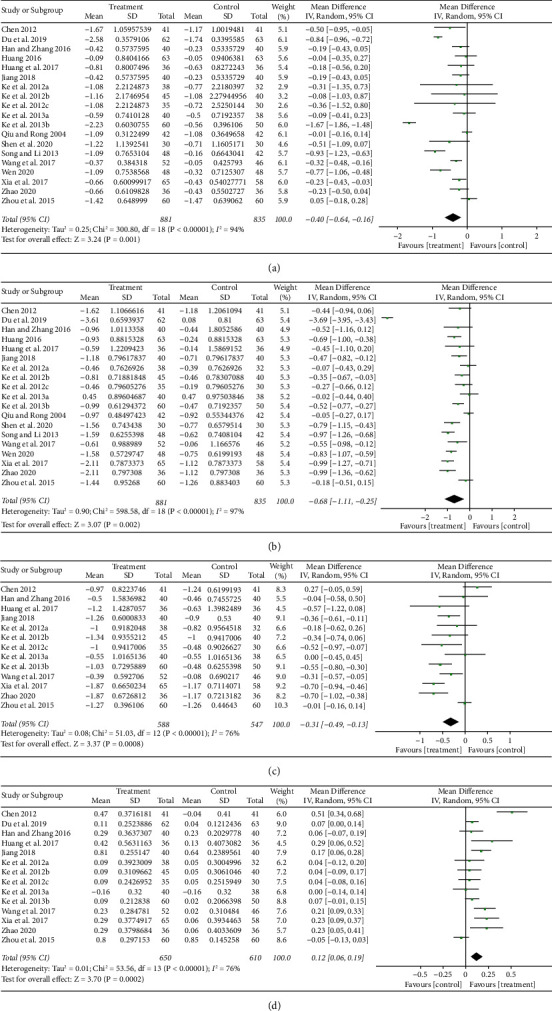
Forest plot of the effects of LGZG on serum lipid parameters of TG (a), TC (b), LDL-c (c), and HDL-c (d).

TG levels were evaluated in the complete 19 studies. The pooled results by random effect models indicated that, LGZG can significantly reduce the level of TG (MD, −0.40 mM; 95% Cl: −0.64 to −0.16; *p*=0.001), with great heterogeneity of 94% (Figure 5(a)). Hence, subgroup analyses were performed using the random effect model, the results of which are shown in Table S5 and Figure S2. Stratification by intervention and control method showed that supplementation with LGZG in combination with WM, compared with WM alone, decreased the level of TG significantly (MD, −0.41 mM, 95% CI:−0.63 to −0.18; *p*=0.0004), whereas LGZG supplementation alone resulted in nonsignificant reduction (*p* > 0.05) compared to no treatment. The subgroup analysis also revealed that supplementation involving long-term treatment (>8 weeks, cut-off by medium value) and led to more reduction in TG (MD, −0.47 mM, 95% CI: −0.82 to −0.12; *p*=0.008) than that achieved with short-term treatment (MD, −0.28 mM, 95% CI: −0.61 to 0.05; *p*=0.10), in which*p*=0.10 indicated potential significant difference when more trials were performed.

The TC levels were also investigated in all 19 studies. The results using random effect model are shown in Figure 5(b), and LGZG significantly reduced the levels of TC (MD, −0.68 mM; 95% Cl: −1.11 to −0.25; *p*=0.002). However, between-study heterogeneity was high (*I*^2^ = 97%). To attenuate the heterogeneity, subgroup analysis was conducted and its results showed that LGZG with or without WM both significantly decreased the level of TC (Table S5 and Figure S3). Supplementation with LGZG in combination with WM showed better effect (MD, −1.07 mM, 95%Cl: −1.98 to −0.16, *p*=0.02) than those of LGZG alone (MD, −0.50 mM, 95% Cl: −0.85 to −0.14, *p*=0.006).

Among the included studies, 13 studies [42–44, 46, 50–55, 57, 58, 60] reported LDL-c indicators and 14 [42–44, 46, 50–55, 57–60] reported HDL-c indicators. Figures 5(c) and 5(d) show that LGZG treatment can significantly reduce LDL-c (MD, −0.31 mM; 95% Cl: −0.49 to −0.13; *p*=0.0008) and increase HDL-c (MD, 0.12 mM; 95% Cl: 0.06 to 0.19; *p*=0.0002), but with great heterogeneity (*I*^*2*^ was 76% for LDL-c and 76% for HDL-c). Results of sensitivity analysis showed that when Chen's research [60] was removed, the heterogeneity of LDL-c and HDL-c decreased slightly (*I*^2^ reducing from 76% to 72% for LDL-c and from 76% to 59% for HDL-c, respectively). Furthermore, the subgroup analysis revealed that supplementation with LGZG in combination with WM, compared with WM alone, could achieve better improvement in LDL-c (MD, −0.32 mM, 95% CI: −0.62 to −0.03; *p*=0.03) and HDL-c (MD, 0.20 mM, 95% CI: 0.10 to 0.30; *p* < 0.0001), than those of supplementation with LGZG alone when compared to no treatment (MD, −0.30 mM, 95% CI: −0.50 to −0.09, *p*=0.005 for LDL-c, and MD, 0.06 mM, 95%CI: −0.02 to 0.13, *p*=0.14 for HDL-c), as shown in Table S5 and Figures S4 and S5.

#### 3.3.3. Effects of LGZG on Obesity Parameters

**Figure 6:**
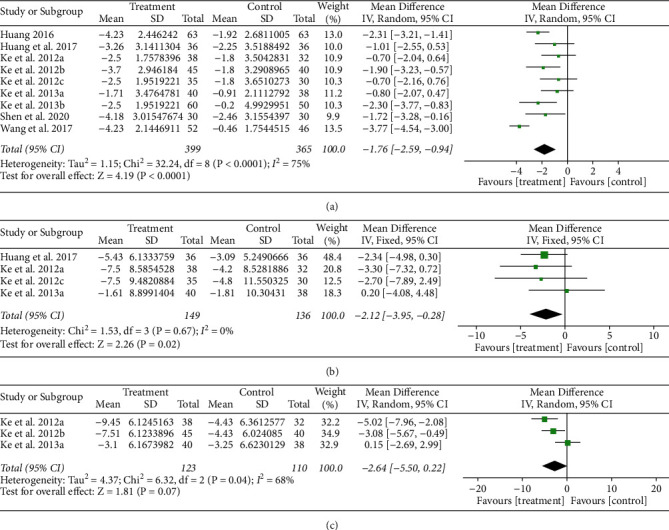
Forest plot of the effects of LGZG on obesity parameters of BMI (a), BW (b), and WC (c).

A total of 9 studies [46, 48, 50–54, 56, 57] of overweight or obese patients assessed the efficacy of LGZG on BMI, and the pooled results showed a significant reduction in the LGZG group compared with the control group (MD, −1.76 kg/m^2^; 95% CI: −2.59, −0.94; *p* < 0.0001). However, between-study heterogeneity was considerably high (*I*^2^ = 75%), as shown in Figure 6(a). Subgroup analyses were performed to determine the principal source of heterogeneity. As shown in Table S5 and Figure S6, pooled results seemed to show that supplementation with LGZG in combination with WM was more effective (MD, −3.77 kg/m^2^; 95% CI: −4.54 to −3.00; *p* < 0.00001) compared with those of LGZG alone (MD,−2.03 kg/m^2^; 95% CI: −3.10 to −0.96; *p* < 0.0002). In the meantime, the effect of LGZG in the studies that implemented short-term treatment (< 8 weeks) was −1.33 kg/m^2^ (95% CI: −2.21 to −0.45; *p* < 0.003), and it was −2.14 kg/m^2^ (95% CI: −3.36 to −0.93; *p*=0.0006) for studies with longer duration (more than 8 weeks). In the sensitivity analyses, the heterogeneity reduced significantly to 0% when two studies was removed [46, 56].

Pooled results of 4 studies [50, 52, 53, 57] concerning BW showed that LGZG brought a reduction of BW by −2.12 kg (95% CI: −3.95 to −0.28; *p*=0.02) compared to the controls, with low heterogeneity (*I*^2^ = 0%) (Figure 6(b)). Three studies [50, 52, 54] also showed possible significant improvement of LGZG effect on WC (MD, −2.64 cm; 95% CI: −5.50 to 0.22; *p*=0.07) (Figure 6(c)). There was no significant change in sensitivity analyses.

### 3.4. Adverse Reactions

An evaluation of six studies [42, 48, 51, 54, 57, 60] revealed no adverse reactions occurring in the clinical therapy. One study [44] reported that there was no statistical adverse reaction rate, another reported [50] minor side effects but no detail, and another two [46, 52] reported the occurrence of adverse reactions including palpitation, headache, nausea, and abdominal discomfort. The remaining nine studies [43, 45, 47, 49, 53, 55, 56, 58, 59] failed to report any adverse effects following clinical treatment. Further systematical assessment on the safety of LGZG is still needed.

### 3.5. Publication Bias Assessment

A funnel plot of LGZG alone or combined with WM compared to clinical control group was applied with RR as the *X*-axis and SE (log RR) for the *Y*-axis. No absolutely symmetrical phenomenon was observed, suggesting there might be some publication bias (data not shown).

Begg's regression analyses were performed to further examine the possibility of publication bias (Figure S7). Results showed that there was no statistically significant publication bias in the analyses of TG, TC, LDL-c, HDL-c, BMI, BW, and WC (*p* > 0.05) except for efficacy rate (*p* < 0.001), which suggested that the pooled result of efficacy rate needs further verification.

### 3.6. Evidence Quality Evaluation by the GRADE Approach

The quality of evidence was evaluated for all outcomes including effective rate, TG, TC, LDL-c, HDL-c, BMI, BW, and WC. Downgrading by one level was due to risk of bias, high heterogeneity (I^2^ > 50%), wide range of 95% confidence interval, or the publication bias tested in Begg's regression, respectively. The results suggested that the certainties of evidence for the effects of LGZG on TG and TC were moderate, and the quality of evidence for other outcomes were low and very low (Table 4).

### 3.7. Potential Mechanism of LGZG for Management of Serum Lipids and Obesity

#### 3.7.1. Active Component Screening

A total of 589 chemical constituents of LGZG were obtained from the TCMSP database. Among them, 120 components of OB ≥ 30% and DL ≥ 0.18 after removing the duplications were listed in Table S6, including 15 compounds in *Poria* (Fu Ling), 7 in *Cinnamomi ramulus* (Gui Zhi), 7 in *Atractylodis macrocephalae rhizoma* (Bai Zhu), and 92 in *Glycyrrhizae radix et rhizoma* (Gan Cao).

#### 3.7.2. Targets Identification and Protein-Protein Interaction (PPI) Network Construction

**Figure 7:**
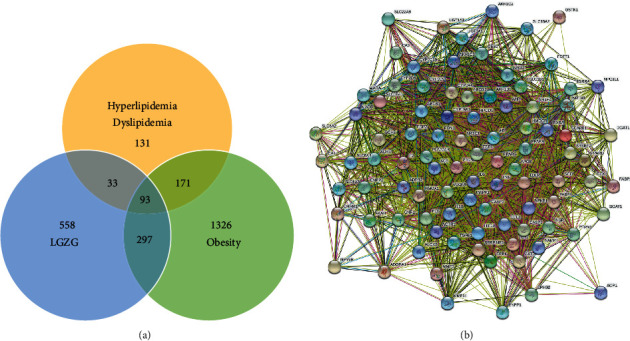
Targets identification of LGZG for obesity and hyperlipidemia/dyslipidemia. (a) Venn diagram; (b) protein-protein interaction network.

A total of 981 targets of LGZG were identified from SEA, SwissTargetPrediction and STITCH database (Figure S8), and 1887 and 428 targets, related to obesity and hyperlipidemia respectively, were obtained from TTD, DrugBank and DisGeNET databases. After matching the targets of LGZG with those related to obesity and hyperlipidemia, 93 potential targets associated with the effect of LGZG for the management of serum lipids and obesity were identified (Figure 7(a) and Table S7). Furthermore, a PPI network was constructed using the STRING database, as shown in Figure 7(b).

#### 3.7.3. GO and KEGG Enrichment Analysis

**Figure 8:**
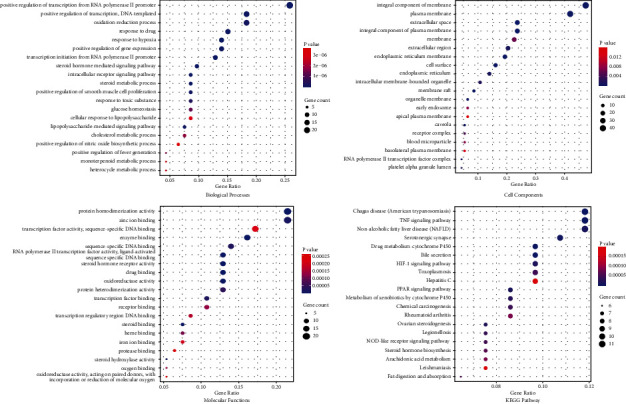
GO and KEGG enrichment analyses of key targets in LGZG for management of serum lipid and obesity (top 20).

To probe into the biological function and potential mechanism of LGZG treatment, GO enrichment analysis of key targets was performed, where 322 significant entries were obtained (*p* < 0.05), including 232 entries for biological processes (BP), 61 for molecular functions (MF), and 29 for cell components (CC). The top 20 entries for BP, MF, and CC are shown in Figures 8(a)–(c), and more details are provided in Table S8. Meanwhile, 56 significant KEGG pathways (*p* < 0.05) associated with the key targets abovementioned were enriched, and the top 20 entries are shown in Figure 8(d) and Table S8.

#### 3.7.4. Bioactive Components Finding by Greedy Algorithms

**Figure 9:**
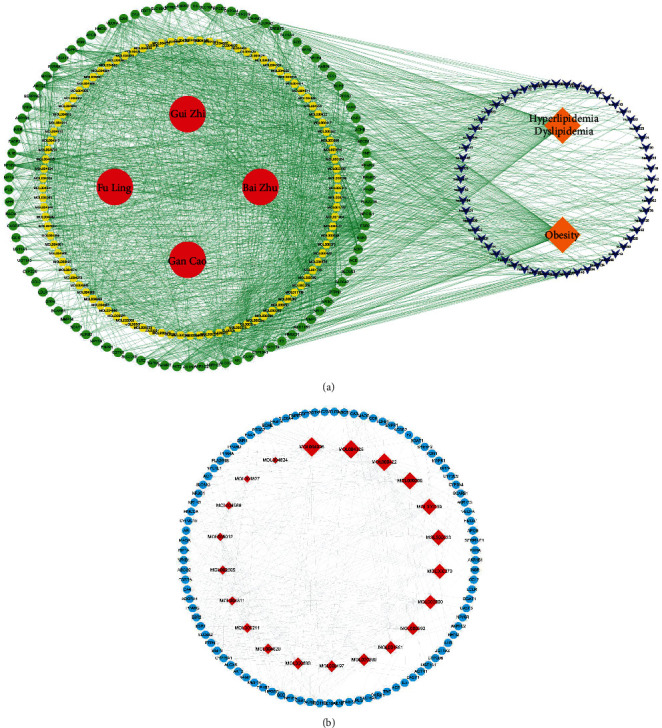
Construction of herb-component-target-pathway network to reveal the regulatory mechanism of LGZG on hyperlipidemia and obesity (a). The red circles, yellow hexagon, and orange diamonds represent the four herbs, active components of LGZG, and diseases, respectively. The green circles represent targets related to LGZG and diseases, and blue V's represent the related pathways. (b) Minimized set of components (red diamond) and targets (cyan circles) network based on greedy algorithms.

Herb-component-target-pathway interaction network was established as illustrated in Figure 9(a). The network consists of 4 herbs, 96 chemical components, 93 protein targets, and 56 KEGG pathways, including 251 nodes and 2148 edges.

**Figure 10:**
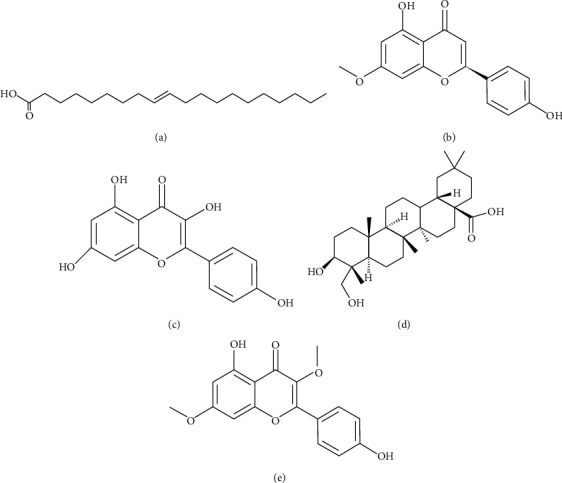
The top five components with higher degree of values in the component-target network, including (a) eicosenoic acid (C_20_H_38_O_2_, molecular weight: 310.5), (b) naringenin (C_15_H_12_O_5_, molecular weight: 272.25), (c) kaempferol (C_15_H_10_O_6_, molecular weight: 286.24), (d) hederagenin (C_30_H_48_O_4_, molecular weight: 472.7), and (e) kumatakenin (C_17_H_14_O_6_, molecular weight: 314.29).5.

Greedy algorithms were applied to find a minimized set of bioactive components of LGZG satisfying cover of all of hub targets. A total of 21 potential bioactive components of LGZG for management of serum lipids and obesity were obtained as shown in Table S9, which mainly involved flavonoids, saponins, and fatty acids. In addition, the network comprising the 21 key components and 93 hub targets was constructed, with a total frequency of 384 (Figure 9(b)). The top 5 targets with higher degree values in the component-target network were PTPN1, CYP19A1, ESR2, AR, and ESR1, and the top 5 components were identified as eicosenoic acid, naringenin, kaempferol, hederagenin, and kumatakenin (Figure 10).

## 4. Discussion

Obesity and hyperlipidemia are commonly linked with an increased risk of many serious cardiovascular diseases [61]. Although LGZG is a promising novel treatment approach for dyslipidemia and obesity [62], evidence regarding its effectiveness is still far from adequate, and the precise mechanisms remain unclarified until now. In the current study, meta-analysis was first conducted to evaluate the clinical value of LGZG for the management of serum lipids and obesity. Also, the bioactive components and potential mechanisms were studied by integrating network pharmacology and greedy algorithms. Results demonstrate that the adjuvant and long-term treatment of LGZG could be a more preferable intervening measure compared with WM for serum lipids and body weight control. Moreover, twenty-one components in LGZG might play a vital role in modulating multiple targets and pathways.

### 4.1. Summary of Evidence

We systematically evaluated the available evidence of LGZG alone, or LGZG combined with WM for the management of serum lipids and obesity. All of the included studies were conducted in China, involving 1716 patients aged from 35 to 70 years with dyslipidemia and/or other metabolic disorders (925 men and 791 women). There were no significant differences in age, sex, or course of the disease between the experiment and control groups. The risks of bias for most of the domains were *low* or *unclear*. Evidence quality evaluated by GRADE showed that the outcomes change in TC and TG were as moderate, suggested that the actual effect is likely to be close to the estimate of effect. The outcomes of effective rate, HDL, LDL, and obesity parameters were rated as low-quality evidence or very low-quality due to risk of bias, high heterogeneity and publication bias, which implied the limited or uncertain effect estimate of LGZG.

The final results could be influenced by the factors of inconsistent interventions and different treatment durations of LGZG in the included studies. To declare with caution, we divided them into three types, namely LGZG vs. no treatment, LGZG plus WM vs. WM, and LGZG vs. WM. Subgroup analyses stratified by whether other interventions along with LGZG seemed to show that LGZG supplement, when used as an adjuvant therapy based on conventional WM, was more effective in improving serum lipids and obesity parameters of TG, TC, LDL-c, HDL-c and BMI, compared with LGZG alone. When compared with WM, however, LGZG supplementation alone resulted in nonsignificant improvement on most of serum lipid parameters such as TG, LDL-c, and HDL-c, except decreased TC and BMI significantly. In addition, the results of subgroup analyses for different treatment durations proved that long-term medication for more than eight weeks was more effective in improving TG, HDL, and BMI. The robustness of our results was confirmed, considering that sensitivity analysis failed to reveal any obvious outliers.

In addition, clinical treatment of TCM was dependent on the diagnosis using syndrome differentiation, which is the key to enhancing the therapeutic effect of treatment. LGZG, as a representative prescription for spleen deficiency syndrome, has the reliable effect of invigorating spleen to damp elimination, activating yang (yang mainly means body function), and promoting diuresis. Due to the factor of lacking syndrome differentiation in most of included trials, subgroup analyses could not be done in this review to investigate whether the selection of inappropriate patients affected the treatment efficacy of LGZG formula. Future RCTs should be recommended to follow the TCM guideline of syndrome differentiation, which can be helpful for improving the quality of trials.

We are supposed to consider the following limitations which could also influence the findings. First, there was a substandard methodological quality of the included trials. Some of them had lacked or just had a brief description of the adequate random allocation method, allocation concealment, or blinding. Second, substantial heterogeneity was observed in most of the pooled outcomes. The reasons for the heterogeneity could be associated with small sample size, different treatment dosage and durations, and inconsistent interventions. The present meta-analysis was lacking in studies with larger sample sizes than 100 participants per group. Third, articles in languages other than English or Chinese have not been included and potential publication bias may exist. Fourth, all of included RCTs were conducted exclusively on Chinese subjects, which may cause the potential racial bias. Fifth, due to the lack of dose-effect relationship evidence, the magnitude of beneficial efficacy of LGZG remained to be clarified. Hence, more rigorous RCTs are demanded to consolidate the clinical evidence.

It is worth mentioning that our protocol was not registered at PROSPERO, this is also an important limitation of this review.

### 4.2. Potential Mechanisms

In traditional Chinese medicine theory, the similar clinic state of dyslipidemia is usually diagnosed as the spleen deficiency syndrome. Among the four herbs of LGZG, *Poria* and *Atractylodis macrocephalae rhizoma* could fortify the spleen and drain dampness, *Cinnamomi ramulus* for assisting yang, and *Glycyrrhizae radix et rhizoma* for dispelling phlegm. A water-insoluble polysaccharide separated from *Poria* significantly improved lipid metabolism and alleviated hepatic steatosis in mice via regulating gut microbes [63]. Flavonoids isolated from *Glycyrrhizae radix et rhizoma* showed the effects of antiobesity and lipid-lowering in the rats fed by high-fat diet [64]. Licochalcone E, a retrochalcone from *Glycyrrhizae radix et rhizoma*, lowered the levels of blood glucose and TG, reduced adipocyte size, and upregulated PPAR*γ* expression in white adipose tissue in the diabetic mice [65]. Besides, the nonaqueous fractions of *G. radix et rhizoma* could have a certain effect on abdominal obesity in diet-induced obese mice [66]. *Atractylodis macrocephalae rhizoma* effectively reduced the adipose tissue weight and serum TG levels, and repaired intestinal epithelial barrier in HFD rats [67]. Atractylenolide I, isolated from *Atractylodis macrocephalae rhizoma*, had an anti-inflammatory effect, possibly related to the NF-*κ*B, ERK1/2, and p38 signaling pathways [68].

Several possible mechanisms for LGZG against both dyslipidemia and obesity have been suggested by the previous studies. LGZG could significantly decrease hepatic triglycerides in HFD rat, probably through increasing serum thyroid hormone levels, and improving beta-oxidation, as well as fatty acid metabolism and transport [11]. LGZG can affect PI3K-Akt and AMPK pathways, and a few targets were found to differentially express such as Pik3r1, Foxo1, Scd1, and Fn1 [69]. LGZG, combined with dietary restriction and regular exercise, decreased the levels of TG, TC, LDL-c, and FFA in rat of metabolic syndrome, possibly due to the inhibition of the serum and liver levels of TNF-*α*, leptin, and PKB [14]. LGZG could also alleviate NAFLD through inhibiting PPP1R3C expression to reduce glycogen synthase activity, promoting glycogen phosphorylase, and reducing glycogen storage [70]. Dang et al. found LGZG treatment could alleviate hepatic steatosis in rats via reducing the m6A methylation levels of SOCS2 [71]. Additionally, LGZG treatment can regulate the oxidative stress-related genes, increasing the expression of antioxidant OSIGN1 and decreasing the expression of AHR which could induce inflammation [13]. Besides, given that PI3K/Akt is a signaling pathway most commonly involved in lipid metabolism in cancer [3], the regulation of cancer metabolism by LGZG could be an interesting topic of future study.

In our study, twenty-one components in LGZG, including naringenin and kaempferol (Figures 10(b)–(c)), were responsible for the effect of management of serum lipids and obesity. And the herb-component-target-pathway network was constructed to reveal the regulatory mechanism of LGZG on hyperlipidemia and obesity first. Previous experiment-based studies supported our finding. Naringenin could increase hepatic fatty acid oxidation, through a PPAR*γ* coactivator 1*α*/PPAR*α*-mediated transcription program [72] Also, naringenin could promote the expression and secretion of adiponectin protein from 3T3-L1 adipocytes [73]. Kaempferol displayed certain obvious antiobesity effects [74, 75], through regulating the gut microbiota [76], inhibiting adipogenesis, and increasing lipolysis [77]. Using the integrated strategy of network pharmacology and greedy algorithms, the important roles of some targets IL6, HMCGR, PPARA, and APOB for management of hyperlipidemia and obesity were highlighted in this work, which was also in accord with the previous publications. IL6 could stimulate lipolysis and fat oxidation in humans [78]. LGZG could markedly inhibit the activity of HMCGR to reduce lipid synthesis in the liver [70]. PPARA plays a role in lipid homeostasis which regulated target genes including lipid metabolism enzymes, lipid transporters, and apolipoproteins [79]. APOB is a major protein constituent of chylomicrons, LDL, and VLDL. Mutation in the gene for APOB will lead to hypercholesterolemia [80]. Besides, CYP3A4 might contribute to cholesterol degradation and bile acid biosynthesis [81]. However, the possible biases to widely studied pathways and functions may influence the predicted results.

## 5. Conclusion

Based on the data mining of 104 cases of the academic experience of famous TCM doctors, this systematic review and meta-analysis about the 19 published RCTs described here indicates that LGZG complementary treatment might be beneficial in improving the serum lipids profile and combating obesity with no significant adverse effects. A panel of active constituents of LGZG, possible targets, and multiple signaling pathways associated with its clinical efficacy were explored. This study provides significant clues for the research on pharmacodynamic material basis and potential mechanism of LGZG in treating obesity and lipid disorders. More rigorous RCTs with larger sample size, as well as biological experiments, are demanded to consolidate the clinical evidence and further elucidate the precise mechanism.

## Figures and Tables

**Table 1:** The characteristics of studies included.

Included studies (authors, year)	Type of intervention	Sample size (T/C)	Sex (M/W)	Average age (year)	Course of disease (year)	Treatment duration	Dosage (form)	Outcomes
Chen (2012)	T : CT + LGZG + WM	41/41	45/37	T:56.53 ± 7.89	T:7.63 ± 3.78	12 weeks	100 ml×2 (D)	TG, TC, LDL-c, HDL-c, FPG, 2h-PG, HbAlc, FINS, HOMA-IR, BMI
	C : CT + WM			C:57.25 ± 6.17	C:7.52 ± 3.61			
Du et al. (2019)	T : LGZG + WM	62/63	73/52	T:59.18 ± 4.62	T:4.69 ± 2.12	1 month	1dose (D)	TC, TG, HDL-c
	C : WM			C:58.27 ± 4.31	C:4.73 ± 2.03			
Han and Zhang (2016)	T : LGZG + WM	40/40	44/36	T:73 ± 1.9	T:4.1 ± 1.8	2 months	4g×3 (G)	TG, TC, LDL-c, HDL-c
	C : WM			C:71 ± 2.3	C:3.7 ± 2			
Huang (2016)	T : CT + LGZG	63/63	79/47	T:37.96 ± 8.89	NA	4 weeks	1dose (D)	TG, TC, SBP, DBP, BMI
	C : CT			C:38.56 ± 6.89				
Huang et al. (2017)	T : CT + LGZG	36/36	39/33	T:43.3 ± 16.21	T:11.32 ± 5.67	8 weeks	150 ml×3 (D)	TG, TC, LDL-c, HDL-c, BMI, BW
	C : CT			C:42.1 ± 17.42	C:10.61 ± 5.56			
Jiang et al. (2018)	T : CT + LGZG + WM	40/40	47/33	T:70.1 ± 8.6	T:12.8 ± 7.5	8 weeks	1dose (D)	TG, TC, LDL-c, HDL-c
	C : CT + WM			C:68.8 ± 7.0	C:11.5 ± 6.7			
Ke et al. (2012a)	T : CT + LGZG	38/32	36/34	T:42.5 ± 8.5	NA	6 months	150 ml×2 (D)	TG, TC, LDL-c, HDL-c, FPG, 2h-PG, FINS, HOMA-IR, BMI, BW,WC
	C : CT			C:42.1 ± 8.2				
Ke et al. (2012b)	T : CT + LGZG	45/40	43/42	T:46.5 ± 7.3	T:3.5 ± 2.4	6 months	1dose (D)	TG, TC, LDL-c, HDL-c, FPG, 2h-PG, HbAlc, FINS, SBP, DBP, BMI, WC
	C : CT			C:45.7 ± 7.5	C:3.8 ± 2.6			
Ke et al. (2012c)	T : CT + LGZG	35/30	30/35	T:45.76 ± 7.14	T:8.2	4 weeks	1dose (D)	TG, TC, LDL-c, HDL-c,
	C : CT			C:46.13 ± 8.73	C:9.4			SBP, DBP, BMI, BW
Ke et al. (2013a)	T : CT + LGZG	40/38	36/42	T:39.39 ± 14.05	NA	1 week	150 ml (D)	TG, TC, LDL-c, HDL-c,
	C : CT			C:38.43 ± 10.12				FPG, BMI, BW, WC
Ke et al. (2013b)	T : CT + LGZG	60/50	52/58	T:41.6 ± 15.34	T:5.8 ± 3.4	3 months	1dose (D)	TG, TC, LDL-c, HDL-c, BMI
	C : CT + WM			C:42.8 ± 14.52	C:5.7 ± 4.5			
Qiu and Rong (2004)	T : LGZG	42/42	50/34	T:52.41 ± 21.40	NA	2 months	200 ml×2 (D)	TG, TC
	C : WM			C:54.23 ± 19.06				
Shen et al. (2020)	T : CT + LGZG	30/30	37/23	T:46.80 ± 10.05	T:4.5	12 weeks	1dose (D)	TG, TC, FPG, FINS, HOMA-IR, BMI
	C : CT + WM			C:46.10 ± 10.16	C:4.0			
Song and Li (2013)	T : LGZG	48/42	48/42	T:44.8 ± 4.2	T:4.24 ± 2.10	3 months	150 ml×2 (D)	TG, TC
	C : WM			C:42.2 ± 4.9	C:4.20 ± 2.12			
Wang et al. (2017)	T : CT + LGZG + WM	52/46	53/45	T:64.33 ± 4.64	NA	12 weeks	150 ml×2 (D)	TG, TC, LDL-c, HDL-c
	C : CT + WM			C:65.6 ± 3.7				SBP, DBP, BMI
Wen (2020)	T : LGZG + WM	48/48	51/45	T:45.69 ± 8.58	T:4.97 ± 1.21	3 months	1dose (D)	TC, TG
	C : WM			C:46.99 ± 9.01	C:4.32 ± 1.28			
Xia et al.(2017)	T : CT + LGZG + WM C : CT + WM	65/58	69/54	T:58.5 ± 11.7	T:3.5 ± 1.6	3 months	150 ml (D)	TG, TC, LDL-c, HDL-c, BMI
				C:57.4 ± 13.5	C:3.3 ± 1.4			
Zhao (2020)	T : CT + LGZG + WM C : CT + WM	36/36	37/35	T:54.63 ± 4.14	NA	1 week	1 dose (D)	TG, TC, LDL-c, HDL-c
				C:53.14 ± 3.28				
Zhou et al. (2015)	T : LGZG	60/60	56/64	T:47.5 ± 6.8	NA	3 months	1 dose (D)	TG, TC, LDL-c, HDL-c
	C : WM			C:46.5 ± 7.5				

T: treatment group; C: control group; M: men; W: women; NA: not available; CT: conventional treatment by nondrug therapy including dietary intervention, fasting, exercise, health education, and others; LGZG : *Ling Gui Zhu Gan* formula; WM: western medicine; D : LGZG decoction; G : LGZG granules; TG: triglyceride; TC: total cholesterol; LDL-c: low-density lipoprotein; HDL-c: high-density lipoprotein; FPG: fasting plasma glucose; 2h-PG:2 hours plasma glucose; HbAlc: glycated hemoglobin; FINS: fasting insulin; HOMA-IR: homeostasis model assessment for insulin resistance; SBP: systolic pressure; DBP: diastolic pressure; BMI: body mass index; BW: body weight; WC: waist circumference.

**Table 2:** The original data of outcome indicators.

Included studies (authors, year	TC (Mm)		TG (Mm)		LDL-c (Mm)		HDL-c (Mm)		BMI (kg/m^2^)		BW (kg)		WC (cm)	
	Baseline	After intervention	Baseline	After intervention	Baseline	After intervention	Baseline	After intervention	Baseline	After intervention	Baseline	After intervention	Baseline	After intervention
Chen (2012)	T:4.81 ± 1.17	T:3.19 ± 1.03	T:3.08 ± 1.22	T:1.41 ± 0.69	T:3.76 ± 0.91	T:2.79 ± 0.69	T:1.09 ± 0.35	T:1.56 ± 0.39	T:25.06 ± 1.42	NA	NA	NA	NA	NA
	C:4.86 ± 1.27	C:3.68 ± 1.13	C:3.06 ± 1.13	C:1.89 ± 0.78	C:3.91 ± 0.66	C:2.67 ± 0.57	C:1.19 ± 0.41	C:1.15 ± 0.41	C:25.15 ± 1.52					

Du et al. (2019)	T:7.42 ± 0.76	T:3.81 ± 0.42	T:3.84 ± 0.41	T:1.26 ± 0.16	NA	NA	T:1.03 ± 0.12	T:1.14 ± 0.29	NA	NA	NA	NA	NA	NA
	C:7.28 ± 0.81	C:7.36 ± 0.81	C:3.72 ± 0.39	C:1.98 ± 0.23			C:1.01 ± 0.13	C:1.05 ± 0.11						

Han and Zhang (2016)	T:5.19 ± 0.98	T:4.23 ± 1.04	T:2.50 ± 0.66	T:2.08 ± 0.38	T:3.09 ± 0.76	T:2.59 ± 0.45	T:0.97 ± 0.42	T:1.26 ± 0.21	NA	NA	NA	NA	NA	NA
	C:5.21 ± 1.32	C:4.77 ± 1.29	C:2.53 ± 0.58	C:2.30 ± 0.47	C:3.12 ± 0.85	C:2.66 ± 0.32	C:0.98 ± 0.18	C:1.21 ± 0.22						

Huang (2016)	T:5.53 ± 0.91	T:4.60 ± 0.85	T:1.55 ± 0.89	T:1.46 ± 0.78	NA	NA	NA	NA	T:27.91 ± 2.64	T:23.68 ± 2.19	NA	NA	NA	NA
	C:5.36 ± 0.85	C:5.12 ± 0.91	C:1.56 ± 0.96	C:1.51 ± 0.92					C:27.88 ± 2.94	C:25.96 ± 2.31				

Huang et al. (2017)	T:5.63 ± 1.33	T:5.04 ± 1.07	T:2.77 ± 0.92	T:1.96 ± 0.54	T:3.89 ± 1.08	T:2.69 ± 1.62	T:1.36 ± 0.34	T:1.78 ± 0.65	T:31.14 ± 3.57	T:27.88 ± 2.34	T:81.25 ± 6.31	T:75.82 ± 5.94	NA	NA
	C:5.45 ± 1.54	C:5.31 ± 1.63	C:2.84 ± 0.79	C:2.21 ± 0.86	C:3.77 ± 1.25	C:3.14 ± 1.51	C:1.42 ± 0.38	C:1.55 ± 0.43	C:30.51 ± 3.09	C:28.26 ± 3.83	C:81.16 ± 5.67	C:79.07 ± 4.69		

Jiang et al. (2018)	T:5.89 ± 0.82	T:4.71 ± 0.77	T:1.84 ± 0.33	T:1.46 ± 0.27	T:3.54 ± 0.64	T:2.28 ± 0.55	T:1.11 ± 0.25	T:1.92 ± 0.26	NA	NA	NA	NA	NA	NA
	C:5.76 ± 0.85	C:5.05 ± 0.78	C:1.83 ± 0.32	C:1.57 ± 0.26	C:3.56 ± 0.53	C:2.66 ± 0.53	C:1.10 ± 0.21	C:1.74 ± 0.26						

Ke et al. (2012a)	T:6.08 ± 0.88	T:5.62 ± 0.41	T:3.49 ± 2.55	T:2.41 ± 1.14	T:3.78 ± 1.06	T:2.78 ± 0.51	T:1.13 ± 0.27	T:1.22 ± 0.45	T:27.5 ± 2.0	T:25.0 ± 1.3	T:78.0 ± 9.9	T:70.5 ± 4.5	T:91.21 ± 7.07	T:81.76 ± 3.39
	C:6.12 ± 0.86	C:5.73 ± 0.58	C:3.41 ± 2.53	C:2.64 ± 1.61	C:3.65 ± 1.08	C:2.83 ± 0.74	C:1.14 ± 0.29	C:1.19 ± 0.31	C:28.5 ± 3.6	C:26.7 ± 2.4	C:76.8 ± 9.6	C:72.6 ± 6.7	C:91.37 ± 7.16	C:86.94 ± 5.00

Qiu and Rong (2004)	T:6.25 ± 0.52	T:5.28 ± 0.44	T:2.64 ± 0.35	T:1.55 ± 0.25	NA	NA	NA	NA	NA	NA	NA	NA	NA	NA
	C:6.18 ± 0.47	C:5.26 ± 0.61	C:2.58 ± 0.42	C:1.50 ± 0.24										

Shen et al. (2020)	T:6.14 ± 0.63	T:4.58 ± 0.82	T:3.46 ± 1.25	T:2.24 ± 0.98	NA	NA	NA	NA	T:27.21 ± 3.15	T:23.03 ± 2.86	NA	NA	NA	NA
	C:6.25 ± 0.57	C:5.48 ± 0.72	C:3.52 ± 1.18	C:2.81 ± 1.14					C:27.53 ± 2.96	C:25.07 ± 3.32				

Song and Li (2013)	T:4.70 ± 0.69	T:3.11 ± 0.53	T:2.21 ± 0.88	T:1.12 ± 0.51	NA	NA	NA	NA	NA	NA	NA	NA	NA	NA
	C:4.58 ± 0.72	C:3.96 ± 0.76	C:2.09 ± 0.76	C:1.93 ± 0.47										

Wang et al. (2017)	T:5.37 ± 1.09	T:4.76 ± 0.84	T:1.65 ± 0.43	T:1.28 ± 0.31	T:3.10 ± 0.64	T:2.71 ± 0.53	T:1.26 ± 0.31	T:1.49 ± 0.25	T:31.92 ± 1.83	T:27.69 ± 2.36	NA	NA	NA	NA
	C:5.29 ± 1.13	C:5.23 ± 1.20	C:1.59 ± 0.47	C:1.54 ± 0.36	C:3.09 ± 0.70	C:3.01 ± 0.68	C:1.29 ± 0.30	C:1.31 ± 0.32	C:31.53 ± 1.64	C:31.07 ± 1.85				

Wen (2020)	T:4.68 ± 0.62	T:3.10 ± 0.51	T:2.18 ± 0.87	T:1.09 ± 0.46	NA	NA	NA	NA	NA	NA	NA	NA	NA	NA
	C:4.70 ± 0.66	C:3.95 ± 0.57	C:2.13 ± 0.81	C:1.81 ± 0.53										

Xia et al. (2017)	T:6.37 ± 0.82	T:4.26 ± 0.75	T:2.23 ± 0.62	T: 1.57 ± 0.58	T:4.19 ± 0.74	T:2.32 ± 0.54	T:1.26 ± 0.35	T:1.55 ± 0.40	T:23.3 ± 2.7	NA	NA	NA	NA	NA
	C:6.44 ± 0.75	C:5.32 ± 0.82	C:2.29 ± 0.55	C:1.86 ± 0.53	C:4.12 ± 0.65	C:2.95 ± 0.76	C:1.22 ± 0.42	C:1.28 ± 0.36	C:22.8 ± 2.2					

Zhao (2020)	T:6.36 ± 0.83	T:4.25 ± 0.76	T:2.22 ± 0.63	T:1.56 ± 0.59	T:4.18 ± 0.75	T:2.31 ± 0.55	T:1.25 ± 0.34	T:1.54 ± 0.41	NA	NA	NA	NA	NA	NA
	C:6.43 ± 0.76	C:5.31 ± 0.83	C:2.28 ± 0.56	C:1.85 ± 0.54	C:4.11 ± 0.66	C:2.94 ± 0.77	C:1.21 ± 0.43	C:1.27 ± 0.37						

Zhou et al. (2015)	T:6.58 ± 1.10	T:5.04 ± 0.52	T:3.10 ± 0.72	T:1.68 ± 0.58	T:2.30 ± 0.43	T:1.03 ± 0.35	T:0.67 ± 0.13	T:1.47 ± 0.34	NA	NA	NA	NA	NA	NA
	C:6.30 ± 1.02	C:5.14 ± 0.54	C:3.12 ± 0.72	C:1.65 ± 0.5	C:2.30 ± 0.51	C:1.04 ± 0.32	C:0.70 ± 0.15	C:1.55 ± 0.14						

**Table 3:** Jadad scoring scales for the included studies by two authors and kappa statistics for methodological quality assessment.

Study	Rater 1	Rater 2	Kappa value	*p*
Chen (2012)	2	2	0.883	<0.001
Du et al. (2019)	3	3		
Han and Zhang (2016)	2	2		
Huang (2016)	1	1		
Huang et al. (2017)	2	2		
Jiang et al. (2018)	3	3		
Ke et al. (2012a)	2	2		
Ke et al. (2012b)	2	2		
Ke et al. (2012c)	3	3		
Ke et al. (2013a)	4	4		
Ke et al. (2013b)	1	3		
Qiu and Rong (2004)	2	2		
Shen et al. (2020)	3	3		
Song and Li (2013)	2	2		
Wang et al. (2017)	2	2		
Wen (2020)	2	2		
Xia et al.(2017)	1	1		
Zhao (2020)	2	2		
Zhou et al. (2015)	4	4		

More details on the Jadad scoring scales are shown in Table S4.

**Table 4:** Grade evidence quality evaluation of included studies.

Outcomes	No. of participants (studies)	Relative effect (95% CI)	Factors that may decrease certainty of evidence					Quality of the evidence (GRADE)
			Risk of bias	Indirectness	Inconsistency	Imprecision	Publication bias	
Effective rate	1169 (13 studies)	1.24 [1.17, 1.32]	Serious^a^	No serious	Not serious	No serious	Serious^d^	⨁⨁◯◯
								Low
TG	1716 (19 studies)	−0.40 [−0.64, −0.16]	Not serious	Serious^b^	Not serious	No serious	None	⨁⨁⨁◯
								Moderate
TC	1716 (19 studies)	−0.68 [−1.11, −0.25]	Not serious	Serious^b^	Not serious	Not serious	None	⨁⨁⨁◯
								Moderate
LDL-c	1135 (13 studies)	−0.31 [−0.49, −0.13]	Serious^a^	Serious^b^	Not serious	Not serious	None	⨁⨁◯◯
								Low
HDL-c	1260 (14 studies)	0.12 [0.06, 0.19]	Serious^a^	Serious^b^	Not serious	Not serious	None	⨁⨁◯◯
								Low
BMI	764 (9 studies)	−1.76 [−2.59, −0.94]	Serious^a^	Serious^b^	Not serious	Serious^c^	None	⨁◯◯◯
								Very low
BW	285 (4 studies)	−2.12 [−3.95, −0.28]	Serious^a^	Not serious	Not serious	Serious^c^	None	⨁⨁◯◯
								Low
WC	233 (3 studies)	−2.64 [−5.50, 0.22]	Serious^a^	Serious^b^	Not serious	Serious^c^	None	⨁◯◯◯
								Very low

^a^This outcome was not reported in all studies; ^b^I^2^ > 50%; ^c^wide range of 95% confidence interval; ^d^*p*<0.001 in Begg's regression analyses.

## Data Availability

All data generated or analyzed during this study are included in the manuscript. The datasets used and/or analyzed during the current study are available from Linjing Zhao (ljzhao@sues.edu.cn) upon reasonable request.

## Abbreviations

BMI: Body mass index
BW: Body weight
HDL-c: High-density lipoprotein cholesterol
LDL-c: Low-density lipoprotein cholesterol
LGZG: *Ling Gui Zhu Gan* formula
RCTs: Randomized controlled trials
TC: Cholesterol
TG: Total triglycerides
WC: Waist circumference
NF-*κ*B: Nuclear factor-kappaB
ERK1/2: Extracellular signal regulated kinase 1/2
p38: p38 mitogen-activated protein kinase
PPAR*γ*: Peroxisome proliferator-activated receptor gamma
C/EBP*α*: CCAAT/enhancer binding protein-alpha
SREBP-1c: Sterol regulatory element-binding protein-1c
TNF-*α*: Tumor necrosis factor-*α*
PKB: Protein kinase B
PPP1R3C: Protein phosphatase 1 regulatory subunit 3C
NAFLD: Nonalcoholic fatty liver disease
m6A: N6-methyladenosine
SOCS2: Suppressor of cytokine signaling 2
OSGIN1: Oxidative stress-induced growth inhibitor 1
TR*β*1: Thyroid hormone receptor03B21
CPT1A: Carnitine palmitoyltransferase-1A
SREBP-1c: Sterol regulatory element-binding protein 1c
ACSL: Long-chain acyl-CoA synthetase
ApoB100: Apolipoprotein B100
PI3K/Akt: Phosphatidylinositol 3- kinase/protein kinase B
AMPK: AMP-activated protein kinase.

## Supplementary Materials

Supplementary Materials The following are available online: *Table S1*. TCM herbs with frequency over 8 in 104 cases reported in the database of National Service Platform for Academic Experience of famous TCM doctors and CNKI, for improvement of hyperlipidemia and obesity. *Table S2*. PRISMA checklist. *Table S3*. Search strategy for Cochrane Library. *Table S4*. Jadad scores of the included studies by two raters. *Table S5*. Subgroup analyses of the effects of LGZG on serum lipids and obesity parameters. *Table S6.* The main active components of LGZG. *Table S7.* Targets of LGZG responsible for treatment of obesity and lipid disorders. *Table S8*. GO and KEGG enrichment analysis of the key targets of LGZG in treating obesity and lipid disorders (top 20). *Table S9.* Bioactive components of LGZG potentially responsible for management of serum lipids and obesity. *Figure S1.* Chemical structures of major components in original or modified LGZG preparation for quality control by HPLC. *Figure S2.* Subgroup analyses for TG according to types of intervention and control and duration of intervention. *Figure S3.* Subgroup analyses for TC according to types of intervention and control. *Figure S4.* Subgroup analyses for LDL-c according to types of intervention and control and duration of intervention. *Figure S5.* Subgroup analyses for HDL-c according to types of intervention and control and duration of intervention. *Figure S6.* Subgroup analyses for BMI according to types of intervention and control and duration of intervention. *Figure S7.* Begg's regression analyses for publication bias. *Figure S8*. Network plot of the active compounds of LGZG and related targets.
