# Supplementary material for: Clinical Evidence and Potential Mechanisms of Complementary Treatment of Ling Gui Zhu Gan Formula for the Management of Serum Lipids and Obesity
Source: Evid Based Complement Alternat Med. 2022 May 9;2022:7714034. doi: 10.1155/2022/7714034 (PMC9110158; doi:10.1155/2022/7714034)
Supplement: Supplementary Materials — The following are available online: Table S1. TCM herbs with frequency over 8 in 104 cases reported in the database of National Service Platform for Academic Experience of famous TCM doctors and CNKI, for improvement of hyperlipidemia and obesity. Table S2. PRISMA checklist. Table S3. Search strategy for Cochrane Library. Table S4. Jadad scores of the included studies by two raters. Table S5. Subgroup analyses of the effects of LGZG on serum lipids and obesity parameters. Table S6. The main active components of LGZG. Table S7. Targets of LGZG responsible for treatment of obesity and lipid disorders. Table S8. GO and KEGG enrichment analysis of the key targets of LGZG in treating obesity and lipid disorders (top 20). Table S9. Bioactive components of LGZG potentially responsible for management of serum lipids and obesity. Figure S1. Chemical structures of major components in original or modified LGZG preparation for quality control by HPLC. Figure S2. Subgroup analyses for TG according to types of intervention and control and duration of intervention. Figure S3. Subgroup analyses for TC according to types of intervention and control. Figure S4. Subgroup analyses for LDL-c according to types of intervention and control and duration of intervention. Figure S5. Subgroup analyses for HDL-c according to types of intervention and control and duration of intervention. Figure S6. Subgroup analyses for BMI according to types of intervention and control and duration of intervention. Figure S7. Begg's regression analyses for publication bias. Figure S8. Network plot of the active compounds of LGZG and related targets. [file 7714034.f1.docx]

**Supporting information**

**Clinical evidence and potential mechanisms of complementary treatment of *Ling Gui Zhu Gan* formula for the management of serum lipids and obesity**

Jiashuai Huang^1^, Linjing Zhao^1*^, Jijia Sun^2^, Lixin Wang^3^, Jianrong Gu^4^, Xijian Liu^1^, Mengwen Yang^1^, Yuting Wang^1^, Ning Zhang^1^, Jiamin Zhu^1^, Shanshan Xu^5^, Xinfeng Ren^1^, Ying Su^6*^

^1^ College of Chemistry and Chemical Engineering, Shanghai University of Engineering Science, Shanghai 201620, China.

^2^ Department of Mathematics and Physics, Pharmacy School, Shanghai University of Traditional Chinese Medicine, Shanghai 201203, China.

^3^ Integrated TCM & Western Medicine Department, Shanghai Pulmonary Hospital Affiliated to Tongji University, Shanghai 200433, China.

^4^ Informatization Office, Shanghai University of Engineering Science, Shanghai 201620, China.

^5^ School of Basic Medical Sciences, Chengdu University of Traditional Chinese Medicine, Chengdu 611137, Sichuan Province, China.

^6^ Department of Pathology, School of Basic Medical Sciences, Fudan University, Shanghai, 200032, China.

**Corresponding authors:**

*****Address correspondence to Dr. Linjing Zhao ([ljzhao@sues.edu.cn](mailto:ljzhao@sues.edu.cn)) and Dr. Ying Su (18111010077@fudan.edu.cn).

**Methods**

**Greedy algorithms for finding a minimal set of bioactive components in LGZG**

**Input:** Small molecule-target relationship list

**Output:** Minimum small molecule set covering all targets

*m* = number of all small molecules

*Targets* = targets of all small molecules

**while** targetNeeded != *NULL* **do:**

bestSmallMolecule = *NULL*

targetCovered = *NULL*

**for** *i* = 1, 2, 3, …, *m* **do**：

Covered = The target set of the *i*-th small molecule ∩ targetNeeded

**if** targetCovered contains targetNeeded **do:**

the *i*-th small molecule = bestSmallMolecule

targetCovered = Covered

**End**

targetNeeded = targetNeeded ∪ targetCovered

Small molecule collection covering all targets = Small molecule collection covering all targets ∪ bestSmallMolecule

**End**

**Table S1.** TCM herbs with frequency over 8 in 104 cases reported in the database of National Service Platform for Academic Experience of Famous TCM doctor and CNKI, for improvement of hyperlipidemia and obesity.

**Table S2**. PRISMA checklist.

**Table S3.** Search strategy for Cochrane Library.

**Table S4**. Jadad scores of the included studies by two raters.

**Table S5.** Subgroup analyses of the effects of LGZG on serum lipids and obesity parameters.

**Table S6.** The main active components of LGZG.

**Table S7.** Targets of LGZG responsible for treatment of obesity and lipid disorders.

**Table S8.** GO and KEGG enrichment analysis of the key targets of LGZG in treating obesity and lipid disorders (Top 20).

**Table S9.** Bioactive components of LGZG potentially responsible for management of serum lipids and obesity.

**Figure S1**. Chemical structures of major components in original or modified LGZG preparation for quality control by HPLC.

**Figure S2**. Subgroup analyses for TG according to types of intervention and control, and duration of intervention.

**Figure S3**. Subgroup analyses for TC according to types of intervention and control.

**Figure S4**. Subgroup analyses for LDL-c according to types of intervention and control, and duration of intervention.

**Figure S5**. Subgroup analyses for HDL-c according to types of intervention and control, and duration of intervention.

**Figure S6**. Subgroup analyses for BMI according to types of intervention and control, and duration of intervention.

**Figure S7**. Bgger's regression analyses for publication bias.

**Figure S8**. Network plot of the active compounds of LGZG and related targets.

**Table S1.** TCM herbs with frequency over 8 in 104 cases reported in the database of National Service Platform for Academic Experience of Famous TCM doctor and CNKI, for improvement of hyperlipidemia and obesity.

| **Chinese name** | | **English name** | **Latin name** | **Latin name of plant** | **Family** |
| --- | --- | --- | --- | --- | --- |
| 山楂 | Shan Zha | Crataegus Pinnatifida | *Crataeft Fructus* | *Crataegus pinnatifida Bge.* | Rosaceae |
| 泽泻 | Ze Xie | Alisma Orientale | *Alismatis Rhizoma* | *Alisma orientalis (Sam.)Juzep.* | Alismataceae |
| 丹参 | Dan Shen | Dan-Shen Root | *Salviae Miltiorrhizae Radix et Rhizoma* | *Salvia miltiorrhiza Bge.* | Lamiaceae |
| 茯苓 | Fu Ling | Indian Buead Tuckahoe | *Poria* | *Poria cocos(Schw.)Wolf* | Polyporaceae |
| 白术 | Bai Zhu | Largehead Atractylodes Rh | *Atractylodis Macrocephalae Rhizoma* | *Atractylodes macrocephala Koidz.* | Compositae |
| 决明子 | Jue Ming Zi | Sickle Senna Seed | *Cssiae Semen* | *Cassia obtusifolia L.* | Leguminosae |
| 川芎 | Chuan Xiong | Sichuan Lovase Rhizome | *Chuanxiong Rhizoma* | *Ligusticum chuanxiong Hort.* | Apiaceae |
| 陈皮 | Chen Pi | Tangerine Peel | *Citri Reticulatae Preicarpium* | *Citrus reticulata Blanco* | Rutaceae |
| 黄芪 | Huang Qi | Milkvetch Root | *Astragali Radix* | *Astragulus embranaceus (Fisch. )Bge.* | Leguminosae |
| 甘草 | Gan Cao | Licorice Root | *Glycyrrhizae Radix et Rhizoma* | *Glycyrrhiza uralensis Fisch.* | Leguminosae |
| 荷叶 | He Ye | Lotus Leaf | *Nelumbinis Folium* | *Nelumbo nucifera Gaertn.* | Nymphaeaceae |
| 党参 | Dang Shen | Tangshen | *Codonopsis Radix* | *Codonopsis pilosula（Franch.）Nannf.* | Campanulaceae |
| 半夏 | Ban Xia | Pinellia Ternata | *Pinelliae Rhizoma* | *Pinellia ternata (Thunb. )Breit.* | Araceae |
| 柴胡 | Chai Hu | Chinese Thorowax Root | *Bupleuri Radix* | *Bupleurum chinense DC.* | Apiaceae |
| 当归 | Dang Gui | Chinese Angelica | *Angelicae Sinensis Radix* | *Angelica sinensis (Oliv.)Diels* | Apiaceae |
| 桂枝 | Gui Zhi | Cassia Twig | *Cinnamomi Ramulus* | *Cinnamomum cassia Presl* | Lauraceae |
| 苍术 | Cang Zhu | Rhizoma Atractylodis | *Atractylodis Rhizoma* | *AtrActylodes lancea (Thunb. )DC.* | Compositae |
| 法半夏 | Fa Ban Xia | Pinellia Tuber | *Pinelliae Rhizoma Praeparatum* | *Pinellia ternata (Thunb. )Breit.* | Araceae |
| 郁金 | Yu Jin | Aromatic Turmeric Root-tuber | *Curcumae Radix* | *Curcuma longa L.* | Zingiberaceae |
| 赤芍 | Chi Shao | Red Peony Root | *Paeoniae Radix Rubra* | *Paeonia veitchii) Lynch* | Ranunculaceae |
| 瓜蒌 | Gua Lou | Mongolian Snakegourd Fruit | *Trichosanthis Fructus* | *Trichosanthes kirilowii Maxim.* | Cueurbitaceae |
| 红花 | Hong Hua | Safflower | *Carthami Flos* | *Carthamus tinctorius L.* | Compositae |
| 何首乌 | He Shou Wu | Tuber Fleeceflower Root | *Polygoni Multiflori Radix* | *Polygonum multiflorum Thunb.* | Polygonaceae |
| 虎杖 | Hu Zhang | Giant Knotweed Rhizome | *Polygoni Cuspidati Rhizoma et Radix* | *Polygonum cuspidatum Sieb. et Zucc.* | Polygonaceae |
| 枳实 | Zhi Shi | Immature Fruit of the Bitter Orange | *Aurantii Fructus Immaturus* | *Citrus aurantium` L.* | Rutaceae |
| 葛根 | Ge Gen | Lobed Kudzuvine Root | *Puerariae Lobatae Radix* | *Pueraria lobata (Willd.) Ohwi* | Leguminosae |
| 石菖蒲 | Shi Chang Pu | Acorus Tatarinowii | *Acori Tatarinowii Rhizoma* | *Acorus tatarinowii Schott* | Araceae |
| 枳壳 | Zhi Qiao | Immature Trifoliate-orange Peel | *Aurantii Fructus* | *Citrus aurantium L.* | Rutaceae |
| 薏苡仁 | Yi Yi Ren | Ma - yuen Jobstears Seed | *Coicis Semen* | *Coix lacryma-jobi L. var. mayuen. (Roman.) Stapf* | Gramineae |
| 三七 | San Qi | Sanchi | *Notoginseng Radix et Rhizoma* | *Panax notoginseng (Burk.) F. H. Chen* | Araliaceae |
| 炙甘草 | Zhi Gan Cao | Radix Glycyrrhizae Preparata | *Glycyrrhizae Radix et Rhizoma Praeparata Cum Melle* | *Glycyrrhiza uralensis Fisch.* | Leguminosae |
| 桃仁 | Tao Ren | Peach Seed | *Persicae Semen* | *Prunus persica (L.) Batsch* | Rosaceae |
| 清半夏 | Qing Ban Xia | Rhizoma Pinelliae Preparata | *Pinelliae Rhizoma Praeparatum Cum Melle* | *Pinellia ternata (Thunb. )Breit.* | Araceae |
| 大黄 | Da Huang | Rhubarb Root and Rhizome | *Rhei Radix et Rhizome* | *Rheum palmatum L.* | Polygonaceae |

**Table S2**. PRISMA checklist

| **Section/topic** | **3##** | **Checklist item** | **Reported or not** |
| --- | --- | --- | --- |
| **TITLE** | | |  |
| Title | 1 | Identify the report as a systematic review, meta-analysis, or both. | YES |
| **ABSTRACT** | | |  |
| Structured summary | 2 | Provide a structured summary including, as applicable: background; objectives; data sources; study eligibility criteria, participants, and interventions; study appraisal and synthesis methods; results; limitations; conclusions and implications of key findings; systematic review registration number. | YES |
| **INTRODUCTION** | | |  |
| Rationale | 3 | Describe the rationale for the review in the context of what is already known. | YES |
| Objectives | 4 | Provide an explicit statement of questions being addressed with reference to participants, interventions, comparisons, outcomes, and study design (PICOS). | YES |
| **METHODS** | | |  |
| Protocol and registration | 5 | Indicate if a review protocol exists, if and where it can be accessed (e.g., Web address), and, if available, provide registration information including registration number. | NO |
| Eligibility criteria | 6 | Specify study characteristics (e.g., PICOS, length of follow-up) and report characteristics (e.g., years considered, language, publication status) used as criteria for eligibility, giving rationale. | YES |
| Information sources | 7 | Describe all information sources (e.g., databases with dates of coverage, contact with study authors to identify additional studies) in the search and date last searched. | YES |
| Search | 8 | Present full electronic search strategy for at least one database, including any limits used, such that it could be repeated. | YES |
| Study selection | 9 | State the process for selecting studies (i.e., screening, eligibility, included in systematic review, and, if applicable, included in the meta-analysis). | YES |
| Data collection process | 10 | Describe method of data extraction from reports (e.g., piloted forms, independently, in duplicate) and any processes for obtaining and confirming data from investigators. | YES |
| Data items | 11 | YES | YES |
| Risk of bias in individual studies | 12 | Describe methods used for assessing risk of bias of individual studies (including specification of whether this was done at the study or outcome level), and how this information is to be used in any data synthesis. | YES |
| Summary measures | 13 | State the principal summary measures (e.g., risk ratio, difference in means). | YES |
| Synthesis of results | 14 | Describe the methods of handling data and combining results of studies, if done, including measures of consistency (e.g., I^2^) for each meta-analysis. | YES |
| **Section/topic** | **3#** | **Checklist item** | **Reported or not** |
| Risk of bias across studies | 15 | Specify any assessment of risk of bias that may affect the cumulative evidence (e.g., publication bias, selective reporting within studies). | YES |
| Additional analyses | 16 | Describe methods of additional analyses (e.g., sensitivity or subgroup analyses, meta-regression), if done, indicating which were pre-specified. | YES |
| **RESULTS** | | |  |
| Study selection | 17 | Give numbers of studies screened, assessed for eligibility, and included in the review, with reasons for exclusions at each stage, ideally with a flow diagram. | YES |
| Study characteristics | 18 | For each study, present characteristics for which data were extracted (e.g., study size, PICOS, follow-up period) and provide the citations. | YES |
| Risk of bias within studies | 19 | Present data on risk of bias of each study and, if available, any outcome level assessment (see item 12). | YES |
| Results of individual studies | 20 | For all outcomes considered (benefits or harms), present, for each study: (a) simple summary data for each intervention group (b) effect estimates and confidence intervals, ideally with a forest plot. | YES |
| Synthesis of results | 21 | Present results of each meta-analysis done, including confidence intervals and measures of consistency. | YES |
| Risk of bias across studies | 22 | Present results of any assessment of risk of bias across studies (see Item 15). | YES |
| Additional analysis | 23 | Give results of additional analyses, if done (e.g., sensitivity or subgroup analyses, meta-regression [see Item 16]). | YES |
| **DISCUSSION** | | |  |
| Summary of evidence | 24 | Summarize the main findings including the strength of evidence for each main outcome; consider their relevance to key groups (e.g., healthcare providers, users, and policy makers). | YES |
| Limitations | 25 | Discuss limitations at study and outcome level (e.g., risk of bias), and at review-level (e.g., incomplete retrieval of identified research, reporting bias). | YES |
| Conclusions | 26 | Provide a general interpretation of the results in the context of other evidence, and implications for future research. | YES |
| **FUNDING** | | |  |
| Funding | 27 | Describe sources of funding for the systematic review and other support (e.g., supply of data); role of funders for the systematic review. | YES |

**Table S3.** Search strategy for Cochrane Library.

| No. | Query results | Results | Date |
| --- | --- | --- | --- |
| #5 | #1 AND #4 | 6 | Dec. 31, 2020 |
| #4 | #2 AND #3 | 27 | Dec. 31, 2020 |
| #3 | obesity OR BMI | 174 | Dec. 31, 2020 |
| #2 | dyslipidemia OR hyperlipidemia OR triglyceride OR total cholesterol OR high density lipoprotein OR low density lipoprotein | 139 | Dec. 31, 2020 |
| #1 | lingguizhugan | 7 | Dec. 31, 2020 |

**Table S4.** The Jadad scores of the included studies by two raters.

| Author | Random sequence generation | Allocation scheme hiding | Blind method | Blind method evaluation of the outcome | Result data integrity | Selective reporting of research findings | Other sources of bias | The Jadad score |
| --- | --- | --- | --- | --- | --- | --- | --- | --- |
| **Rater 1** |  |  |  |  |  |  |  |  |
| Chen (2012) | The order of treatment, high | Dimness | Dimness | Dimness | No data for BMI after intervention | Low | Low | 2 |
| Du et al. (2019) | Random number table, low | Yes, low | Dimness | Dimness | Compete, low | The outcome of lipid was incomplete | Low | 3 |
| Han and Zhang (2016) | The order of treatment, high | Dimness | Dimness | Dimness | Compete, low | Low | Low | 2 |
| Huang (2016) | The order of treatment, high | Dimness | Dimness | Dimness | Compete, low | The outcome of lipid was incomplete | Low | 1 |
| Huang et al. (2017) | Dimness | Dimness | Dimness | Dimness | Compete, low | Low | Low | 2 |
| Jiang et al. (2018) | Random number table, low | Yes, low | Dimness | Dimness | Compete, low | Low | Low | 3 |
| Ke et al. (2012a) | Dimness | Dimness | Dimness | Dimness | Compete, low | Low | Low | 2 |
| Ke et al. (2012b) | Dimness | Dimness | Dimness | Dimness | Compete, low | Low | Fasting was different between treatment group and the control group | 2 |
| Ke et al. (2012c) | Random number table, low | Yes, low | Dimness | Dimness | Compete, low | Low | Fasting was different between treatment group and the control group | 3 |
| Ke et al. (2013a) | Random number table, low | Yes, low | Dimness | Dimness | Compete, low | Low | Low | 4 |
| Ke et al. (2013b) | The order of treatment, high | Yes | Dimness | Dimness | Compete, low | Low | Fasting was different between treatment group and the control group | 1 |
| Qiu and Rong (2004) | Dimness | Dimness | Dimness | Dimness | Compete, low | The outcome of lipid was incomplete | Low | 2 |
| Shen et al. (2020) | Random number table, low | Yes, low | Dimness | Dimness | Compete, low | The outcome of lipid was incomplete | Fasting was different between treatment group and the control group | 3 |
| Song and Li (2013) | Dimness | Dimness | Dimness | Dimness | Compete, low | The outcome of lipid was incomplete | Low | 2 |
| Wang et al. (2017) | Dimness | Dimness | Dimness | Dimness | Compete, low | Low | Low | 2 |
| Wen (2020) | The order of therapeutic schedule, high | Dimness | Dimness | Dimness | Compete, low | The outcome of lipid was incomplete | Low | 2 |
| Xia et al.(2017) | The order of treatment, high | Dimness | Dimness | Dimness | No data for BMI after intervention | Low | Low | 1 |
| Zhao (2020) | Yes, Dimness | Dimness | Dimness | Dimness | Compete, low | Low | Low | 2 |
| Zhou et al. (2015) | Random number table, low | Yes, low | Dimness | Dimness | Compete, low | Low | Low | 4 |
| **Rater 2** |  |  |  |  |  |  |  |  |
| Chen (2012) | The order of treatment, high | Dimness | Dimness | Dimness | No data for BMI after intervention | Low | Low | 2 |
| Du et al. (2019) | Random number table, low | Yes, low | Dimness | Dimness | Compete, low | The outcome of lipid was incomplete | Low | 3 |
| Han and Zhang (2016) | The order of treatment, high | Dimness | Dimness | Dimness | Compete, low | Low | Low | 2 |
| Huang (2016) | The order of treatment, high | Dimness | Dimness | Dimness | Compete, low | The outcome of lipid was incomplete | Low | 1 |
| Huang et al. (2017) | Dimness | Dimness | Dimness | Dimness | Compete, low | Low | Low | 2 |
| Jiang et al. (2018) | Random number table, low | Yes, low | Dimness | Dimness | Compete, low | Low | Low | 3 |
| Ke et al. (2012a) | Dimness | Dimness | Dimness | Dimness | Compete, low | Low | Low | 2 |
| Ke et al. (2012b) | Dimness | Dimness | Dimness | Dimness | Compete, low | Low | Fasting was different between treatment group and the control group | 2 |
| Ke et al. (2012c) | Random number table, low | Yes, low | Dimness | Dimness | Compete, low | Low | Fasting was different between treatment group and the control group | 3 |
| Ke et al. (2013a) | Random number table, low | Yes, low | Dimness | Dimness | Compete,low | Low | Low | 4 |
| Ke et al. (2013b) | The order of treatment, high | Yes | Dimness | Dimness | Compete,low | Low | Fasting was different between treatment group and the control group | 3 |
| Qiu and Rong (2004) | Dimness | Dimness | Dimness | Dimness | Compete, low | The outcome of lipid was incomplete | Low | 2 |
| Shen et al. (2020) | Random number table, low | Yes, low | Dimness | Dimness | Compete, low | The outcome of lipid was incomplete | Fasting was different between treatment group and the control group | 3 |
| Song and Li (2013) | Dimness | Dimness | Dimness | Dimness | Compete, low | The outcome of lipid was incomplete | Low | 2 |
| Wang et al. (2017) | Dimness | Dimness | Dimness | Dimness | Compete, low | Low | Low | 2 |
| Wen (2020) | The order of therapeutic schedule, high | Dimness | Dimness | Dimness | Compete, low | The outcome of lipid was incomplete | Low | 2 |
| Xia et al.(2017) | The order of treatment, high | Dimness | Dimness | Dimness | No data for BMI after intervention | Low | Low | 1 |
| Zhao (2020) | Yes, Dimness | Dimness | Dimness | Dimness | Compete, low | Low | Low | 2 |
| Zhou et al. (2015) | Random number table, low | Yes, low | Dimness | Dimness | Compete, low | Low | Low | 4 |

**Table S5.** Subgroup analyses of the effects of LGZG on serum lipids and obesity parameters.

| **Outcome** | **Pooled effect size (95%CI)** | **P*** | ***I*^2^ (%)** |
| --- | --- | --- | --- |
| **TG** |  |  |  |
| **Intervention and control method** |  |  |  |
| LGZG vs no treatment | -0.11 (-0.29, 0.08) | 0.25 | 0 |
| LGZG plus western medicine vs western medicine | -0.41 (-0.63, -0.18) | 0.0004 | 89 |
| LGZG vs western medicine | -0.62 (-1.38, 0.15) | 0.12 | 98 |
| **Duration** |  |  |  |
| ≤8 weeks | -0.28 (-0.61, 0.05) | 0.10 | 89 |
| ＞8 weeks | -0.47 (-0.82, -0.12) | 0.008 | 95 |
|  |  |  |  |
| **TC** |  |  |  |
| **Intervention and control method** |  |  |  |
| LGZG vs no treatment | -0.31 (-0.53, -0.10) | 0.005 | 48 |
| LGZG plus western medicine vs western medicine | -1.07 (-1.98, -0.16) | 0.02 | 98 |
| LGZG vs western medicine | -0.50 (-0.85, -0.14) | 0.006 | 87 |
| **Duration** |  |  |  |
| ≤8 weeks | -0.95 (-2.11, -0.22) | 0.11 | 99 |
| ＞8 weeks | -0.53 (-0.74, -0.31) | <0.00001 | 81 |
|  |  |  |  |
| **LDL-c** |  |  |  |
| **Intervention and control method** |  |  |  |
| LGZG vs no treatment | -0.30 (-0.50, -0.09) | 0.005 | 0 |
| LGZG plus western medicine vs western medicine | -0.32 (-0.62, -0.03) | 0.03 | 82 |
| LGZG vs western medicine | -0.27 (-0.80, 0.26) | 0.32 | 92 |
| **Duration** |  |  |  |
| ≤8 weeks | -0.43 (-0.66, -0.20) | 0.0002 | 41 |
| ＞8 weeks | -0.24 (-0.48, -0.00) | 0.05 | 82 |
|  |  |  |  |
| **HDL-c** |  |  |  |
| **Intervention and control method** |  |  |  |
| LGZG vs no treatment | 0.06 (-0.02, 0.13) | 0.14 | 17 |
| LGZG plus western medicine vs western medicine | 0.20 (0.10, 0.30) | <0.0001 | 78 |
| LGZG vs western medicine | 0.01 (-0.11, 0.13) | 0.86 | 76 |
| **Duration** |  |  |  |
| ≤8 weeks | 0.11 (0.04, 0.18) | 0.003 | 49 |
| ＞8 weeks | 0.13 (0.03, 0.23) | 0.01 | 84 |
|  |  |  |  |
| **BMI:** |  |  |  |
| **Intervention and control method** |  |  |  |
| LGZG vs no treatment | -1.35 (-1.99, -0.71) | ＜0.0001 | 34 |
| LGZG plus western medicine vs western medicine | -3.77 (-4.54, -3.00) | ＜0.00001 | NA |
| LGZG vs western medicine | -2.03 (-3.10, -0.96) | 0.0002 | 0 |
| **Duration (cut-off: medium)** |  |  |  |
| ≤8 weeks | -1.33 (-2.21, -0.45) | 0.003 | 49 |
| ＞8 weeks | -2.14 (-3.36, -0.93) | 0.0006 | 79 |

**Table S6.** The main active components of LGZG.

| **Molecule name** | **Molecule ID** | **OB (%)** | **DL** |
| --- | --- | --- | --- |
| (2R)-2-[(3S,5R,10S,13R,14R,16R,17R)-3,16-dihydroxy-4,4,10,13,14-pentamethyl-2,3,5,6,12,15,16,17-octahydro-1H-cyclopenta[a]phenanthren-17-yl]-6-methylhept-5-enoic acid | MOL000273 | 30.93 | 0.81 |
| trametenolic acid | MOL000275 | 38.71 | 0.8 |
| 7,9(11)-dehydropachymic acid | MOL000276 | 35.11 | 0.81 |
| Cerevisterol | MOL000279 | 37.96 | 0.77 |
| (2R)-2-[(3S,5R,10S,13R,14R,16R,17R)-3,16-dihydroxy-4,4,10,13,14-pentamethyl-2,3,5,6,12,15,16,17-octahydro-1H-cyclopenta[a]phenanthren-17-yl]-5-isopropyl-hex-5-enoic acid | MOL000280 | 31.07 | 0.82 |
| ergosta-7,22E-dien-3beta-ol | MOL000282 | 43.51 | 0.72 |
| Ergosterol peroxide | MOL000283 | 40.36 | 0.81 |
| (2R)-2-[(5R,10S,13R,14R,16R,17R)-16-hydroxy-3-keto-4,4,10,13,14-pentamethyl-1,2,5,6,12,15,16,17-octahydrocyclopenta[a]phenanthren-17-yl]-5-isopropyl-hex-5-enoic acid | MOL000285 | 38.26 | 0.82 |
| 3beta-Hydroxy-24-methylene-8-lanostene-21-oic acid | MOL000287 | 38.7 | 0.81 |
| pachymic acid | MOL000289 | 33.63 | 0.81 |
| Poricoic acid A | MOL000290 | 30.61 | 0.76 |
| Poricoic acid B | MOL000291 | 30.52 | 0.75 |
| poricoic acid C | MOL000292 | 38.15 | 0.75 |
| hederagenin | MOL000296 | 36.91 | 0.75 |
| dehydroeburicoic acid | MOL000300 | 44.17 | 0.83 |
| (-)-taxifolin | MOL001736 | 60.51 | 0.27 |
| beta-sitosterol | MOL000358 | 36.91 | 0.75 |
| (+)-catechin | MOL000492 | 54.83 | 0.24 |
| ent-Epicatechin | MOL000073 | 48.96 | 0.24 |
| taxifolin | MOL004576 | 57.84 | 0.27 |
| Peroxyergosterol | MOL011169 | 44.39 | 0.82 |
| 12-senecioyl-2E,8E,10E-atractylentriol | MOL000020 | 62.4 | 0.22 |
| 14-acetyl-12-senecioyl-2E,8E,10E-atractylentriol | MOL000021 | 60.31 | 0.31 |
| 14-acetyl-12-senecioyl-2E,8Z,10E-atractylentriol | MOL000022 | 63.37 | 0.3 |
| α-Amyrin | MOL000028 | 39.51 | 0.76 |
| (24S)-24-Propylcholesta-5-ene-3beta-ol | MOL000033 | 36.23 | 0.78 |
| 3β-acetoxyatractylone | MOL000049 | 54.07 | 0.22 |
| 8β-ethoxy atractylenolide Ⅲ | MOL000072 | 35.95 | 0.21 |
| Inermine | MOL001484 | 75.18 | 0.54 |
| DFV | MOL001792 | 32.76 | 0.18 |
| Mairin | MOL000211 | 55.38 | 0.78 |
| Glycyrol | MOL002311 | 90.78 | 0.67 |
| kumatakenin | MOL000239 | 50.83 | 0.29 |
| Medicarpin | MOL002565 | 49.22 | 0.34 |
| isorhamnetin | MOL000354 | 49.6 | 0.31 |
| sitosterol | MOL000359 | 36.91 | 0.75 |
| Lupiwighteone | MOL003656 | 51.64 | 0.37 |
| 7-Methoxy-2-methyl isoflavone | MOL003896 | 42.56 | 0.2 |
| formononetin | MOL000392 | 69.67 | 0.21 |
| Calycosin | MOL000417 | 47.75 | 0.24 |
| kaempferol | MOL000422 | 41.88 | 0.24 |
| naringenin | MOL004328 | 59.29 | 0.21 |
| (2S)-2-[4-hydroxy-3-(3-methylbut-2-enyl)phenyl]-8,8-dimethyl-2,3-dihydropyrano[2,3-f]chromen-4-one | MOL004805 | 31.79 | 0.72 |
| euchrenone | MOL004806 | 30.29 | 0.57 |
| glyasperin B | MOL004808 | 65.22 | 0.44 |
| glyasperin F | MOL004810 | 75.84 | 0.54 |
| Glyasperin C | MOL004811 | 45.56 | 0.4 |
| Isotrifoliol | MOL004814 | 31.94 | 0.42 |
| (E)-1-(2,4-dihydroxyphenyl)-3-(2,2-dimethylchromen-6-yl)prop-2-en-1-one | MOL004815 | 39.62 | 0.35 |
| kanzonols W | MOL004820 | 50.48 | 0.52 |
| (2S)-6-(2,4-dihydroxyphenyl)-2-(2-hydroxypropan-2-yl)-4-methoxy-2,3-dihydrofuro[3,2-g]chromen-7-one | MOL004824 | 60.25 | 0.63 |
| Semilicoisoflavone B | MOL004827 | 48.78 | 0.55 |
| Glepidotin A | MOL004828 | 44.72 | 0.35 |
| Glepidotin B | MOL004829 | 64.46 | 0.34 |
| Phaseolinisoflavan | MOL004833 | 32.01 | 0.45 |
| Glypallichalcone | MOL004835 | 61.6 | 0.19 |
| 8-(6-hydroxy-2-benzofuranyl)-2,2-dimethyl-5-chromenol | MOL004838 | 58.44 | 0.38 |
| Licochalcone B | MOL004841 | 76.76 | 0.19 |
| licochalcone G | MOL004848 | 49.25 | 0.32 |
| 3-(2,4-dihydroxyphenyl)-8-(1,1-dimethylprop-2-enyl)-7-hydroxy-5-methoxy-coumarin | MOL004849 | 59.62 | 0.43 |
| Licoricone | MOL004855 | 63.58 | 0.47 |
| Gancaonin A | MOL004856 | 51.08 | 0.4 |
| Gancaonin B | MOL004857 | 48.79 | 0.45 |
| licorice glycoside E | MOL004860 | 32.89 | 0.27 |
| 3-(3,4-dihydroxyphenyl)-5,7-dihydroxy-8-(3-methylbut-2-enyl)chromone | MOL004863 | 66.37 | 0.41 |
| 5,7-dihydroxy-3-(4-methoxyphenyl)-8-(3-methylbut-2-enyl)chromone | MOL004864 | 30.49 | 0.41 |
| 2-(3,4-dihydroxyphenyl)-5,7-dihydroxy-6-(3-methylbut-2-enyl)chromone | MOL004866 | 44.15 | 0.41 |
| Glycyrin | MOL004879 | 52.61 | 0.47 |
| Licocoumarone | MOL004882 | 33.21 | 0.36 |
| Licoisoflavone | MOL004883 | 41.61 | 0.42 |
| Licoisoflavone B | MOL004884 | 38.93 | 0.55 |
| licoisoflavanone | MOL004885 | 52.47 | 0.54 |
| shinpterocarpin | MOL004891 | 80.3 | 0.73 |
| (E)-3-[3,4-dihydroxy-5-(3-methylbut-2-enyl)phenyl]-1-(2,4-dihydroxyphenyl)prop-2-en-1-one | MOL004898 | 46.27 | 0.31 |
| liquiritin | MOL004903 | 65.69 | 0.74 |
| licopyranocoumarin | MOL004904 | 80.36 | 0.65 |
| 3,22-Dihydroxy-11-oxo-delta(12)-oleanene-27-alpha-methoxycarbonyl-29-oic acid | MOL004905 | 34.32 | 0.55 |
| Glyzaglabrin | MOL004907 | 61.07 | 0.35 |
| Glabridin | MOL004908 | 53.25 | 0.47 |
| Glabranin | MOL004910 | 52.9 | 0.31 |
| Glabrene | MOL004911 | 46.27 | 0.44 |
| Glabrone | MOL004912 | 52.51 | 0.5 |
| 1,3-dihydroxy-9-methoxy-6-benzofurano[3,2-c]chromenone | MOL004913 | 48.14 | 0.43 |
| 1,3-dihydroxy-8,9-dimethoxy-6-benzofurano[3,2-c]chromenone | MOL004914 | 62.9 | 0.53 |
| Eurycarpin A | MOL004915 | 43.28 | 0.37 |
| glycyroside | MOL004917 | 37.25 | 0.79 |
| (-)-Medicocarpin | MOL004924 | 40.99 | 0.95 |
| Sigmoidin-B | MOL004935 | 34.88 | 0.41 |
| (2R)-7-hydroxy-2-(4-hydroxyphenyl)chroman-4-one | MOL004941 | 71.12 | 0.18 |
| (2S)-7-hydroxy-2-(4-hydroxyphenyl)-8-(3-methylbut-2-enyl)chroman-4-one | MOL004945 | 36.57 | 0.32 |
| Isoglycyrol | MOL004948 | 44.7 | 0.84 |
| Isolicoflavonol | MOL004949 | 45.17 | 0.42 |
| HMO | MOL004957 | 38.37 | 0.21 |
| 1-Methoxyphaseollidin | MOL004959 | 69.98 | 0.64 |
| Quercetin der. | MOL004961 | 46.45 | 0.33 |
| 3'-Hydroxy-4'-O-Methylglabridin | MOL004966 | 43.71 | 0.57 |
| licochalcone a | MOL000497 | 40.79 | 0.29 |
| 3'-Methoxyglabridin | MOL004974 | 46.16 | 0.57 |
| 2-[(3R)-8,8-dimethyl-3,4-dihydro-2H-pyrano[6,5-f]chromen-3-yl]-5-methoxyphenol | MOL004978 | 36.21 | 0.52 |
| Inflacoumarin A | MOL004980 | 39.71 | 0.33 |
| icos-5-enoic acid | MOL004985 | 30.7 | 0.2 |
| Kanzonol F | MOL004988 | 32.47 | 0.89 |
| 6-prenylated eriodictyol | MOL004989 | 39.22 | 0.41 |
| 7,2',4'-trihydroxy－5-methoxy-3－arylcoumarin | MOL004990 | 83.71 | 0.27 |
| 7-Acetoxy-2-methylisoflavone | MOL004991 | 38.92 | 0.26 |
| 8-prenylated eriodictyol | MOL004993 | 53.79 | 0.4 |
| gadelaidic acid | MOL004996 | 30.7 | 0.2 |
| Vestitol | MOL000500 | 74.66 | 0.21 |
| Gancaonin G | MOL005000 | 60.44 | 0.39 |
| Gancaonin H | MOL005001 | 50.1 | 0.78 |
| Licoagrocarpin | MOL005003 | 58.81 | 0.58 |
| Glyasperins M | MOL005007 | 72.67 | 0.59 |
| Glycyrrhiza flavonol A | MOL005008 | 41.28 | 0.6 |
| Licoagroisoflavone | MOL005012 | 57.28 | 0.49 |
| 18α-hydroxyglycyrrhetic acid | MOL005013 | 41.16 | 0.71 |
| Odoratin | MOL005016 | 49.95 | 0.3 |
| Phaseol | MOL005017 | 78.77 | 0.58 |
| Xambioona | MOL005018 | 54.85 | 0.87 |
| dehydroglyasperins C | MOL005020 | 53.82 | 0.37 |
| quercetin | MOL000098 | 46.43 | 0.28 |

OB, oral bioavailability; DL, drug-likeness.

**Table S7.** Targets of LGZG potentially responsible for treatment of obesity and lipid disorders.

| **Uniprot ID** | **Gene symbol** | **Degree** | **Gene name** |
| --- | --- | --- | --- |
| P05231 | IL6 | 82 | Interleukin-6 |
| P37231 | PPARG | 81 | Peroxisome proliferator-activated receptor gamma |
| P01375 | TNF | 81 | Tumor necrosis factor |
| P03372 | ESR1 | 80 | Estrogen receptor |
| P35354 | PTGS2 | 77 | Prostaglandin G/H synthase 2 |
| P01584 | IL1B | 75 | Interleukin-1 beta |
| P04150 | NR3C1 | 74 | Glucocorticoid receptor |
| P15692 | VEGFA | 74 | Vascular endothelial growth factor A |
| P42574 | CASP3 | 74 | Caspase-3 |
| P04035 | HMGCR | 73 | 3-hydroxy-3-methylglutaryl-coenzyme A reductase |
| P12821 | ACE | 72 | Angiotensin-converting enzyme |
| P28482 | MAPK1 | 71 | Mitogen-activated protein kinase 1 |
| P04114 | APOB | 70 | Apolipoprotein B-100 |
| Q07869 | PPARA | 69 | Peroxisome proliferator-activated receptor alpha |
| P08684 | CYP3A4 | 69 | Cytochrome P450 3A4 |
| P01130 | LDLR | 69 | Low-density lipoprotein receptor |
| P10275 | AR | 68 | Androgen receptor |
| O60760 | HPGDS | 68 | Hematopoietic prostaglandin D synthase |
| P13500 | CCL2 | 68 | C-C motif chemokine 2 |
| Q96RI1 | NR1H4 | 67 | Bile acid receptor |
| P08183 | ABCB1 | 65 | ATP-dependent translocase ABCB1 |
| P11511 | CYP19A1 | 64 | Aromatase |
| P09601 | HMOX1 | 63 | Heme oxygenase 1 |
| Q9UNQ0 | ABCG2 | 63 | Broad substrate specificity ATP-binding cassette transporter ABCG2 |
| O75469 | NR1I2 | 60 | Nuclear receptor subfamily 1 group I member 2 |
| P01137 | TGFB1 | 60 | Transforming growth factor beta-1 proprotein |
| P07550 | ADRB2 | 59 | Beta-2 adrenergic receptor |
| O60603 | TLR2 | 59 | Toll-like receptor 2 |
| P15090 | FABP4 | 58 | Fatty acid-binding protein, adipocyte |
| O00767 | SCD | 57 | Acyl-CoA desaturase |
| P05121 | SERPINE1 | 57 | Plasminogen activator inhibitor 1 |
| P04054 | PLA2G1B | 56 | Phospholipase A2 |
| Q16665 | HIF1A | 56 | Hypoxia-inducible factor 1-alpha |
| P00747 | PLG | 55 | Plasminogen |
| P35869 | AHR | 55 | Aryl hydrocarbon receptor |
| P21554 | CNR1 | 54 | Cannabinoid receptor 1 |
| Q12772 | SREBF2 | 54 | Sterol regulatory element-binding protein 2 |
| P27487 | DPP4 | 53 | Dipeptidyl peptidase 4 |
| P22303 | ACHE | 52 | Acetylcholinesterase |
| P30556 | AGTR1 | 51 | Type-1 angiotensin II receptor |
| P00734 | F2 | 51 | Prothrombin |
| P28845 | HSD11B1 | 51 | Corticosteroid 11-beta-dehydrogenase isozyme 1 |
| Q92731 | ESR2 | 51 | Estrogen receptor beta |
| P06276 | BCHE | 51 | Cholinesterase |
| P15121 | AKR1B1 | 51 | Aldo-keto reductase family 1 member B1 |
| P10635 | CYP2D6 | 50 | Cytochrome P450 2D6 |
| Q8WTV0 | SCARB1 | 50 | Scavenger receptor class B member 1 |
| P33261 | CYP2C19 | 49 | Cytochrome P450 2C19 |
| Q03181 | PPARD | 49 | Peroxisome proliferator-activated receptor delta |
| P11712 | CYP2C9 | 49 | Cytochrome P450 2C9 |
| P19793 | RXRA | 49 | Retinoic acid receptor RXR-alpha |
| Q13133 | NR1H3 | 48 | Oxysterols receptor LXR-alpha |
| P23141 | CES1 | 47 | Liver carboxylesterase 1 |
| P27169 | PON1 | 46 | Serum paraoxonase/arylesterase 1 |
| P06213 | INSR | 46 | Insulin receptor |
| P19838 | NFKB1 | 46 | Nuclear factor NF-kappa-B p105 subunit |
| P09917 | ALOX5 | 45 | Arachidonate 5-lipoxygenase |
| P35557 | GCK | 45 | Hexokinase-4 |
| P43490 | NAMPT | 44 | Nicotinamide phosphoribosyltransferase |
| P31751 | AKT2 | 44 | RAC-beta serine/threonine-protein kinase |
| P29466 | CASP1 | 43 | Caspase-1 |
| P08254 | MMP3 | 43 | Stromelysin-1 |
| P18031 | PTPN1 | 42 | Tyrosine-protein phosphatase non-receptor type 1 |
| P27338 | MAOB | 42 | Amine oxidase [flavin-containing] B |
| P21397 | MAOA | 39 | Amine oxidase [flavin-containing] A |
| P22309 | UGT1A1 | 38 | UDP-glucuronosyltransferase 1-1 |
| Q01959 | SLC6A3 | 37 | Sodium-dependent dopamine transporter |
| P31645 | SLC6A4 | 37 | Sodium-dependent serotonin transporter |
| P30542 | ADORA1 | 37 | Adenosine receptor A1 |
| P42330 | AKR1C3 | 36 | Aldo-keto reductase family 1 member C3 |
| O75907 | DGAT1 | 33 | Diacylglycerol O-acyltransferase 1 |
| O00519 | FAAH | 32 | Fatty-acid amide hydrolase 1 |
| P37268 | FDFT1 | 31 | Squalene synthase |
| P12104 | FABP2 | 31 | Fatty acid-binding protein, intestinal |
| P11474 | ESRRA | 30 | Steroid hormone receptor ERR1 |
| P35503 | UGT1A3 | 29 | UDP-glucuronosyltransferase 1-3 |
| P50281 | MMP14 | 29 | Matrix metalloproteinase-14 |
| P35610 | SOAT1 | 29 | Sterol O-acyltransferase 1 |
| Q12908 | SLC10A2 | 28 | Ileal sodium/bile acid cotransporter |
| Q9UHC9 | NPC1L1 | 27 | NPC1-like intracellular cholesterol transporter 1 |
| Q13822 | ENPP2 | 27 | Ectonucleotide pyrophosphatase/phosphodiesterase family member 2 |
| P07451 | CA3 | 25 | Carbonic anhydrase 3 |
| P22413 | ENPP1 | 25 | Ectonucleotide pyrophosphatase/phosphodiesterase family member 1 |
| P29323 | EPHB2 | 24 | Ephrin type-B receptor 2 |
| P53985 | SLC16A1 | 23 | Monocarboxylate transporter 1 |
| P22748 | CA4 | 23 | Carbonic anhydrase 4 |
| P52895 | AKR1C2 | 22 | Aldo-keto reductase family 1 member C2 |
| P23975 | SLC6A2 | 22 | Sodium-dependent noradrenaline transporter |
| P11229 | CHRM1 | 21 | Muscarinic acetylcholine receptor M1 |
| Q8TCC7 | SLC22A8 | 15 | Solute carrier family 22 member 8 |
| Q15761 | NPY5R | 9 | Neuropeptide Y receptor type 5 |
| P24666 | ACP1 | 8 | Low molecular weight phosphotyrosine protein phosphatase |
| Q9Y2Q3 | GSTK1 | 6 | Glutathione S-transferase kappa 1 |

**Table S8.** GO and KEGG enrichment analyses of the key targets of LGZG in treating obesity and lipid disorders (Top 20).

| **ID** | **Term** | **Count** | **%** | **P** | **Genes** |
| --- | --- | --- | --- | --- | --- |
| **GO enrichment analysis**  ***Biological Processes*** | | | | | |
| GO:0001666 | response to hypoxia | 13 | 13.98 | 1.62E-10 | TGFB1, HIF1A, SLC6A4, VEGFA, DPP4, MMP14, CASP3, CASP1, ADORA1, CCL2, HMOX1, PPARA, TLR2 |
| GO:0006367 | transcription initiation from RNA polymerase II promoter | 12 | 12.90 | 6.65E-10 | ESRRA, AR, RXRA, NR1I2, NR1H4, NR1H3, PPARG, PPARA, NR3C1, ESR1, ESR2, PPARD |
| GO:0043401 | steroid hormone mediated signaling pathway | 9 | 9.68 | 7.90E-10 | ESRRA, RXRA, NR1I2, NR1H4, NR1H3, PPARG, PPARA, ESR1, PPARD |
| GO:0030522 | intracellular receptor signaling pathway | 8 | 8.60 | 1.29E-09 | ESRRA, AR, NR1I2, NR1H4, NR1H3, AHR, PPARA, PPARD |
| GO:0045944 | positive regulation of transcription from RNA polymerase II promoter | 24 | 25.81 | 1.95E-09 | ESRRA, TGFB1, PLA2G1B, NR1I2, SERPINE1, NR1H4, NR1H3, AHR, ADRB2, NR3C1, HIF1A, ESR1, TNF, SREBF2, NFKB1, VEGFA, AR, IL6, RXRA, IL1B, NAMPT, PPARG, PPARA, TLR2 |
| GO:0008202 | steroid metabolic process | 8 | 8.60 | 3.23E-09 | CYP2C9, UGT1A1, CYP2D6, NR1I2, AKR1C3, AKR1C2, CYP2C19, CYP3A4 |
| GO:0042493 | response to drug | 14 | 15.05 | 1.08E-08 | BCHE, TGFB1, ABCB1, MAOB, UGT1A1, PTGS2, SLC6A2, SLC6A3, SLC6A4, IL6, NPC1L1, CASP3, PPARG, ABCG2 |
| GO:0045893 | positive regulation of transcription, DNA-templated | 17 | 18.28 | 1.78E-08 | TGFB1, INSR, NR1I2, NR1H3, AHR, HIF1A, ESR1, TNF, ESR2, NFKB1, AR, IL6, IL1B, MAPK1, PPARG, PPARA, PPARD |
| GO:0031663 | lipopolysaccharide-mediated signaling pathway | 7 | 7.53 | 1.85E-08 | SCARB1, TGFB1, IL1B, MAPK1, CCL2, TNF, TLR2 |
| GO:0010628 | positive regulation of gene expression | 13 | 13.98 | 1.94E-08 | TGFB1, NR1I2, HIF1A, TNF, SLC6A4, VEGFA, AR, IL6, IL1B, APOB, LDLR, TLR2, PPARD |
| GO:0048661 | positive regulation of smooth muscle cell proliferation | 8 | 8.60 | 3.58E-08 | IL6, NPY5R, NAMPT, AKR1B1, HMOX1, HMGCR, PTGS2, TNF |
| GO:0055114 | oxidation-reduction process | 17 | 18.28 | 1.22E-07 | GSTK1, MAOB, MAOA, AKR1C3, AKR1B1, AKR1C2, HMGCR, CYP3A4, PTGS2, CYP2C19, CYP19A1, HSD11B1, CYP2C9, SCD, CYP2D6, ALOX5, FDFT1 |
| GO:0009636 | response to toxic substance | 8 | 8.60 | 4.10E-07 | MAOB, PON1, MAPK1, AHR, SLC22A8, SLC6A4, TLR2, CES1 |
| GO:0042593 | glucose homeostasis | 8 | 8.60 | 1.33E-06 | IL6, SLC16A1, CNR1, INSR, NR1H4, PPARG, HIF1A, GCK |
| GO:0031622 | positive regulation of fever generation | 4 | 4.30 | 1.58E-06 | CNR1, IL1B, PTGS2, TNF |
| GO:0008203 | cholesterol metabolic process | 7 | 7.53 | 1.91E-06 | RXRA, SOAT1, PON1, APOB, LDLR, SREBF2, PPARD |
| GO:0071222 | cellular response to lipopolysaccharide | 8 | 8.60 | 2.83E-06 | IL6, SERPINE1, NR1H4, CCL2, NR1H3, TNF, NFKB1, PPARD |
| GO:0046483 | heterocycle metabolic process | 4 | 4.30 | 3.15E-06 | UGT1A1, CYP2D6, CYP2C19, CYP3A4 |
| GO:0016098 | monoterpenoid metabolic process | 4 | 4.30 | 3.15E-06 | CYP2C9, CYP2D6, CYP2C19, CYP3A4 |
| GO:0045429 | positive regulation of nitric oxide biosynthetic process | 6 | 6.45 | 3.61E-06 | IL6, IL1B, INSR, PTGS2, ESR1, TNF |
| ***Molecular Functions*** | | | | | |
| GO:0004879 | RNA polymerase II transcription factor activity, ligand-activated sequence-specific DNA binding | 12 | 12.90 | 3.61E-17 | ESRRA, AR, RXRA, NR1I2, NR1H4, NR1H3, PPARG, AHR, PPARA, ESR1, ESR2, PPARD |
| GO:0003707 | steroid hormone receptor activity | 12 | 12.90 | 8.25E-15 | ESRRA, AR, RXRA, NR1I2, NR1H4, NR1H3, PPARG, PPARA, NR3C1, ESR1, ESR2, PPARD |
| GO:0008144 | drug binding | 12 | 12.90 | 2.92E-13 | CYP2C9, ACE, CHRM1, CNR1, NPC1L1, CYP2D6, NAMPT, NR1I2, PPARG, PPARA, SLC6A3, PPARD |
| GO:0019899 | enzyme binding | 15 | 16.13 | 3.24E-09 | PTPN1, BCHE, TGFB1, UGT1A1, CYP3A4, PTGS2, CYP2C19, HIF1A, ESR1, ESR2, AR, RXRA, HMOX1, UGT1A3, PPARG |
| GO:0005496 | steroid binding | 7 | 7.53 | 6E-09 | ESRRA, AR, UGT1A1, CYP3A4, NR3C1, ESR1, ESR2 |
| GO:0042803 | protein homodimerization activity | 20 | 21.51 | 1.12E-08 | ACHE, SCARB1, TGFB1, SLC16A1, MAOB, UGT1A1, PON1, HMGCR, ADRB2, PTGS2, NFKB1, SLC6A4, VEGFA, DPP4, HPGDS, NAMPT, ENPP1, HMOX1, UGT1A3, ABCG2 |
| GO:0016491 | oxidoreductase activity | 12 | 12.90 | 1.14E-08 | HSD11B1, CYP2C9, MAOB, MAOA, CYP2D6, SCD, AKR1C3, AKR1B1, AKR1C2, CYP2C19, CYP3A4, FDFT1 |
| GO:0008395 | steroid hydroxylase activity | 5 | 5.38 | 1.13E-05 | CYP2C9, CYP2D6, CYP2C19, CYP3A4, CYP19A1 |
| GO:0008270 | zinc ion binding | 20 | 21.51 | 1.43E-05 | ESRRA, PTPN1, ACE, NR1I2, MMP3, NR1H4, NR1H3, NR3C1, ESR1, ESR2, AR, MMP14, RXRA, CA3, CA4, ENPP2, ENPP1, PPARG, PPARA, PPARD |
| GO:0043565 | sequence-specific DNA binding | 13 | 13.98 | 2.39E-05 | ESRRA, NR1I2, NR1H4, NR1H3, NR3C1, HIF1A, ESR1, ESR2, AR, RXRA, PPARG, PPARA, PPARD |
| GO:0008134 | transcription factor binding | 10 | 10.75 | 2.44E-05 | AR, ENPP2, MAPK1, PPARG, AHR, PPARA, HIF1A, ESR1, NFKB1, PPARD |
| GO:0046982 | protein heterodimerization activity | 12 | 12.90 | 4.44E-05 | TGFB1, RXRA, UGT1A1, ADORA1, AGTR1, UGT1A3, AHR, HIF1A, NFKB1, TLR2, PPARD, VEGFA |
| GO:0020037 | heme binding | 7 | 7.53 | 0.000103 | CYP2C9, CYP2D6, HMOX1, CYP2C19, CYP3A4, PTGS2, CYP19A1 |
| GO:0019825 | oxygen binding | 5 | 5.38 | 0.000123 | CYP2C9, CYP2D6, CYP2C19, CYP3A4, CYP19A1 |
| GO:0005102 | receptor binding | 10 | 10.75 | 0.00013 | DPP4, GSTK1, AR, PLA2G1B, SERPINE1, CCL2, PLG, EPHB2, F2, SLC6A3 |
| GO:0044212 | transcription regulatory region DNA binding | 8 | 8.60 | 0.000163 | AR, RXRA, NR1H3, PPARG, AHR, TNF, SREBF2, NFKB1 |
| GO:0005506 | iron ion binding | 7 | 7.53 | 0.000189 | CYP2C9, CYP2D6, SCD, ALOX5, CYP2C19, CYP3A4, CYP19A1 |
| GO:0003700 | transcription factor activity, sequence-specific DNA binding | 16 | 17.20 | 0.000204 | ESRRA, NR1I2, NR1H4, NR1H3, AHR, NR3C1, HIF1A, ESR1, SREBF2, ESR2, NFKB1, AR, RXRA, PPARG, PPARA, PPARD |
| GO:0002020 | protease binding | 6 | 6.45 | 0.000226 | DPP4, CASP3, SERPINE1, TNF, LDLR, SLC6A3 |
| GO:0016705 | oxidoreductase activity, acting on paired donors, with incorporation or reduction of molecular oxygen | 5 | 5.38 | 0.000262 | CYP2C9, CYP2D6, CYP2C19, CYP3A4, CYP19A1 |
| ***Cell Components*** | | | | | |
| GO:0009986 | cell surface | 15 | 16.13 | 5.37E-07 | ACHE, SCARB1, TGFB1, ABCB1, PLA2G1B, PLG, SLC6A2, TNF, SLC6A3, VEGFA, DPP4, CA4, ENPP1, LDLR, TLR2 |
| GO:0005789 | endoplasmic reticulum membrane | 18 | 19.35 | 1.26E-06 | FAAH, UGT1A1, DGAT1, HMGCR, CYP3A4, PTGS2, CYP2C19, CYP19A1, SREBF2, HSD11B1, CYP2C9, SOAT1, SCD, CYP2D6, HMOX1, UGT1A3, APOB, FDFT1 |
| GO:0005615 | extracellular space | 22 | 23.66 | 2.66E-06 | ACHE, TGFB1, ACE, PLA2G1B, PON1, MMP3, SERPINE1, AKR1B1, PLG, F2, TNF, VEGFA, IL6, IL1B, ALOX5, NAMPT, ENPP2, CCL2, ENPP1, HMOX1, APOB, CES1 |
| GO:0005887 | integral component of plasma membrane | 22 | 23.66 | 5.76E-06 | SCARB1, SLC16A1, CHRM1, UGT1A1, NPY5R, SLC10A2, INSR, ADRB2, SLC6A2, TNF, SLC6A3, SLC6A4, MMP14, CNR1, ADORA1, ENPP2, AGTR1, ENPP1, EPHB2, SLC22A8, LDLR, TLR2 |
| GO:0005886 | plasma membrane | 39 | 41.94 | 4.61E-05 | ACHE, SCARB1, CHRM1, ABCB1, SERPINE1, PLG, ADRB2, SLC6A2, TNF, SLC6A3, SLC6A4, DPP4, CNR1, NPC1L1, CASP3, AKT2, CA4, ADORA1, ENPP2, HMOX1, ENPP1, EPHB2, APOB, LDLR, PTPN1, TGFB1, ACE, SLC16A1, NPY5R, SLC10A2, INSR, F2, ESR1, AR, MMP14, AGTR1, SLC22A8, ABCG2, TLR2 |
| GO:0031090 | organelle membrane | 6 | 6.45 | 7.83E-05 | CYP2C9, FAAH, CYP2D6, CYP2C19, CYP3A4, PTGS2 |
| GO:0045121 | membrane raft | 8 | 8.60 | 8.28E-05 | DPP4, CNR1, CASP3, SLC6A2, TNF, SLC6A3, SLC6A4, TLR2 |
| GO:0016021 | integral component of membrane | 44 | 47.31 | 0.00013 | ACHE, SCARB1, FAAH, ABCB1, MAOB, MAOA, HMGCR, ADRB2, CYP3A4, SLC6A2, TNF, CYP19A1, SLC6A3, SLC6A4, DPP4, HSD11B1, CYP2D6, CNR1, NPC1L1, CA4, ADORA1, ENPP2, HMOX1, ENPP1, UGT1A3, EPHB2, LDLR, ACP1, FDFT1, PTPN1, BCHE, ACE, SLC16A1, UGT1A1, DGAT1, ESR1, AR, MMP14, SOAT1, SCD, AGTR1, SLC22A8, ABCG2, TLR2 |
| GO:0005901 | caveola | 5 | 5.38 | 0.000325 | SCARB1, INSR, HMOX1, MAPK1, PTGS2 |
| GO:0090575 | RNA polymerase II transcription factor complex | 4 | 4.30 | 0.000609 | RXRA, NR1H3, PPARG, HIF1A |
| GO:0005783 | endoplasmic reticulum | 13 | 13.98 | 0.000917 | PTPN1, UGT1A1, HMGCR, PTGS2, CYP19A1, SREBF2, SOAT1, SCD, CYP2D6, ADORA1, HMOX1, UGT1A3, FDFT1 |
| GO:0005576 | extracellular region | 19 | 20.43 | 0.001025 | ACHE, BCHE, TGFB1, ACE, PLA2G1B, PON1, MMP3, SERPINE1, PLG, F2, TNF, ESR2, VEGFA, IL6, IL1B, CASP1, CCL2, EPHB2, APOB |
| GO:0043231 | intracellular membrane-bounded organelle | 10 | 10.75 | 0.00202 | GSTK1, SCARB1, CYP2C9, CNR1, INSR, PON1, PPARG, APOB, CYP2C19, CYP3A4 |
| GO:0031093 | platelet alpha granule lumen | 4 | 4.30 | 0.002702 | TGFB1, SERPINE1, PLG, VEGFA |
| GO:0043235 | receptor complex | 5 | 5.38 | 0.003918 | RXRA, INSR, NR1H3, ADRB2, LDLR |
| GO:0005769 | early endosome | 6 | 6.45 | 0.006085 | PTPN1, AKT2, MAPK1, ADRB2, APOB, LDLR |
| GO:0016020 | membrane | 21 | 22.58 | 0.006107 | GSTK1, ACHE, BCHE, ACE, SLC16A1, CHRM1, ABCB1, INSR, SLC6A2, ESR1, CYP19A1, TNF, SREBF2, VEGFA, DPP4, HSD11B1, SOAT1, SCD, CA4, HMOX1, LDLR |
| GO:0072562 | blood microparticle | 5 | 5.38 | 0.007368 | BCHE, TGFB1, PON1, PLG, F2 |
| GO:0016323 | basolateral plasma membrane | 5 | 5.38 | 0.013113 | CA4, ADORA1, ENPP1, SLC22A8, LDLR |
| GO:0016324 | apical plasma membrane | 6 | 6.45 | 0.015979 | DPP4, ABCB1, NPC1L1, SLC10A2, CA4, ADRB2 |
| **KEGG Pathway Enrichment Analysis** | | | | | |
| hsa05142 | Chagas disease (American trypanosomiasis) | 11 | 11.83 | 3.92E-07 | MAPK1, ACE, IL6, TNF, CCL2, SERPINE1, TLR2, IL1B, NFKB1, TGFB1, AKT2 |
| hsa04668 | TNF signaling pathway | 11 | 11.83 | 5.12E-07 | MAPK1, CASP3, IL6, TNF, CCL2, PTGS2, IL1B, NFKB1, MMP14, MMP3, AKT2 |
| hsa00982 | Drug metabolism - cytochrome P450 | 9 | 9.68 | 1.31E-06 | CYP3A4, UGT1A3, CYP2C19, CYP2C9, MAOA, GSTK1, MAOB, CYP2D6, UGT1A1 |
| hsa04976 | Bile secretion | 9 | 9.68 | 1.47E-06 | LDLR, HMGCR, RXRA, SLC22A8, SCARB1, ABCB1, SLC10A2, NR1H4, ABCG2 |
| hsa04726 | Serotonergic synapse | 10 | 10.75 | 6.49E-06 | MAPK1, CASP3, CYP2C19, PTGS2, CYP2C9, MAOA, SLC6A4, MAOB, CYP2D6, ALOX5 |
| hsa04932 | Non-alcoholic fatty liver disease (NAFLD) | 11 | 11.83 | 1.19E-05 | PPARA, CASP3, IL6, TNF, RXRA, IL1B, NFKB1, INSR, TGFB1, AKT2, NR1H3 |
| hsa03320 | PPAR signaling pathway | 8 | 8.60 | 1.4E-05 | PPARA, PPARD, RXRA, SCD, PPARG, FABP4, FABP2, NR1H3 |
| hsa04066 | HIF-1 signaling pathway | 9 | 9.68 | 1.79E-05 | MAPK1, IL6, HIF1A, HMOX1, VEGFA, SERPINE1, NFKB1, INSR, AKT2 |
| hsa04913 | Ovarian steroidogenesis | 7 | 7.53 | 2.38E-05 | AKR1C3, LDLR, PTGS2, SCARB1, ALOX5, INSR, CYP19A1 |
| hsa00980 | Metabolism of xenobiotics by cytochrome P450 | 8 | 8.60 | 2.7E-05 | CYP3A4, AKR1C2, UGT1A3, CYP2C9, GSTK1, CYP2D6, HSD11B1, UGT1A1 |
| hsa05134 | Legionellosis | 7 | 7.53 | 4.18E-05 | CASP3, IL6, TNF, TLR2, IL1B, NFKB1, CASP1 |
| hsa05204 | Chemical carcinogenesis | 8 | 8.60 | 4.5E-05 | CYP3A4, UGT1A3, CYP2C19, PTGS2, CYP2C9, GSTK1, HSD11B1, UGT1A1 |
| hsa05145 | Toxoplasmosis | 9 | 9.68 | 4.83E-05 | MAPK1, CASP3, TNF, LDLR, TLR2, NFKB1, ALOX5, TGFB1, AKT2 |
| hsa04621 | NOD-like receptor signaling pathway | 7 | 7.53 | 5.16E-05 | MAPK1, IL6, TNF, CCL2, IL1B, NFKB1, CASP1 |
| hsa00140 | Steroid hormone biosynthesis | 7 | 7.53 | 6.31E-05 | AKR1C3, CYP3A4, AKR1C2, UGT1A3, HSD11B1, UGT1A1, CYP19A1 |
| hsa05323 | Rheumatoid arthritis | 8 | 8.60 | 8.33E-05 | IL6, TNF, CCL2, VEGFA, TLR2, IL1B, MMP3, TGFB1 |
| hsa00590 | Arachidonic acid metabolism | 7 | 7.53 | 8.4E-05 | AKR1C3, CYP2C19, PTGS2, CYP2C9, PLA2G1B, ALOX5, HPGDS |
| hsa04975 | Fat digestion and absorption | 6 | 6.45 | 9.45E-05 | APOB, DGAT1, PLA2G1B, NPC1L1, SCARB1, FABP2 |
| hsa05160 | Hepatitis C | 9 | 9.68 | 0.000184 | MAPK1, PPARA, TNF, LDLR, RXRA, SCARB1, NFKB1, AKT2, NR1H3 |
| hsa05140 | Leishmaniasis | 7 | 7.53 | 0.000197 | MAPK1, TNF, PTGS2, TLR2, IL1B, NFKB1, TGFB1 |

**Table S9.** Bioactive components of LGZG potentially responsible for management of serum lipids and obesity.

| **Molecule ID** | **Molecule name** | **OB (%)** | **DL** | **Degree** |
| --- | --- | --- | --- | --- |
| MOL004996 | eicosenoic acid | 30.7 | 0.2 | 33 |
| MOL004328 | naringenin | 59.29 | 0.21 | 29 |
| MOL000422 | kaempferol | 41.88 | 0.24 | 28 |
| MOL000296 | hederagenin | 36.91 | 0.75 | 26 |
| MOL000239 | kumatakenin | 50.83 | 0.29 | 24 |
| MOL000033 | (24S)-24-Propylcholesta-5-ene-3beta-ol | 36.23 | 0.78 | 23 |
| MOL000279 | cerevisterol | 37.96 | 0.77 | 22 |
| MOL005000 | gancaonin G | 60.44 | 0.39 | 21 |
| MOL004991 | 7-acetoxy-2-methylisoflavone | 38.92 | 0.26 | 20 |
| MOL000392 | formononetin | 69.67 | 0.21 | 20 |
| MOL000280 | (2R)-2-[(3S,5R,10S,13R,14R,16R,17R)-3,16-dihydroxy-4,4,10,13,14-pentamethyl-2,3,5,6,12,15,16,17-octahydro-1H-cyclopenta[a]phenanthren-17-yl]-5-isopropyl-hex-5-enoic acid | 31.07 | 0.82 | 18 |
| MOL000497 | licochalcone A | 40.79 | 0.29 | 17 |
| MOL000300 | dehydroeburicoic acid | 44.17 | 0.83 | 17 |
| MOL004820 | kanzonols W | 50.48 | 0.52 | 16 |
| MOL000211 | mairin | 55.38 | 0.78 | 14 |
| MOL004811 | glyasperin C | 45.56 | 0.4 | 12 |
| MOL002565 | medicarpin | 49.22 | 0.34 | 11 |
| MOL005012 | licoagroisoflavone | 57.28 | 0.49 | 10 |
| MOL004966 | 3'-hydroxy-4'-O-methylglabridin | 43.71 | 0.57 | 10 |
| MOL004827 | semilicoisoflavone B | 48.78 | 0.55 | 7 |
| MOL004824 | (2S)-6-(2,4-dihydroxyphenyl)-2-(2-hydroxypropan-2-yl)-4-methoxy-2,3-dihydrofuro[3,2-g]chromen-7-one | 60.25 | 0.63 | 6 |


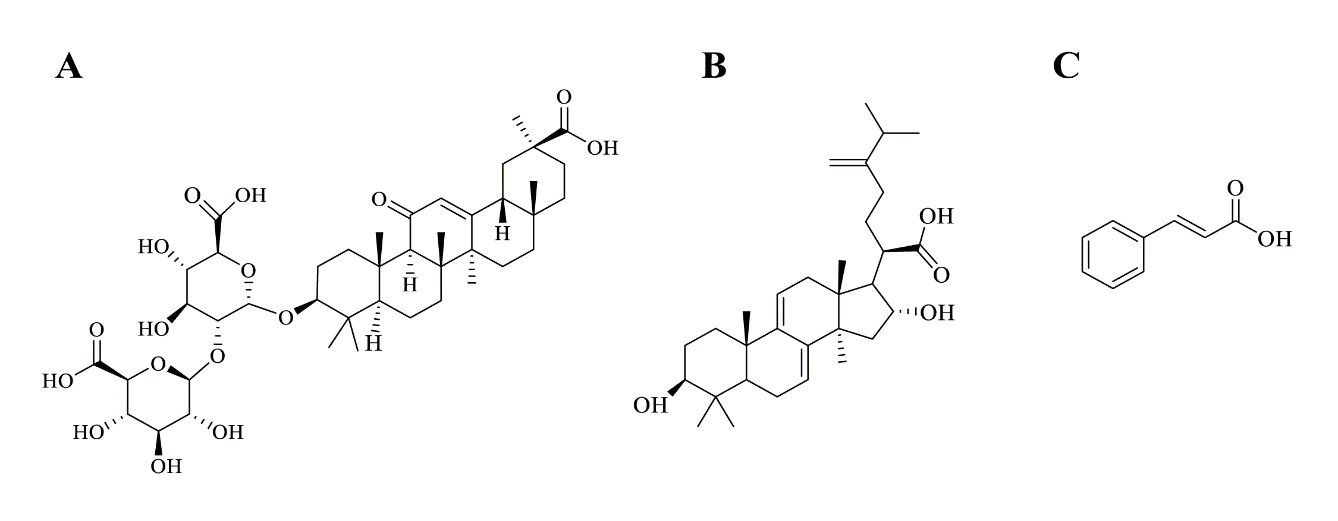


**Figure S1**. Chemical structures of major components in original or modified LGZG preparation for quality control by HPLC[[1-3](#_ENREF_1)]^,^ including (A) glycyrrhizic acid (C_42_H_62_O_16_, molecular weight: 822.93), (B) dehydrotumulosic acid (C_31_H_48_O_4_, molecular weight: 484.71) and (C) cinnamic acid (C_9_H_8_O_2_, molecular weight: 148.16).


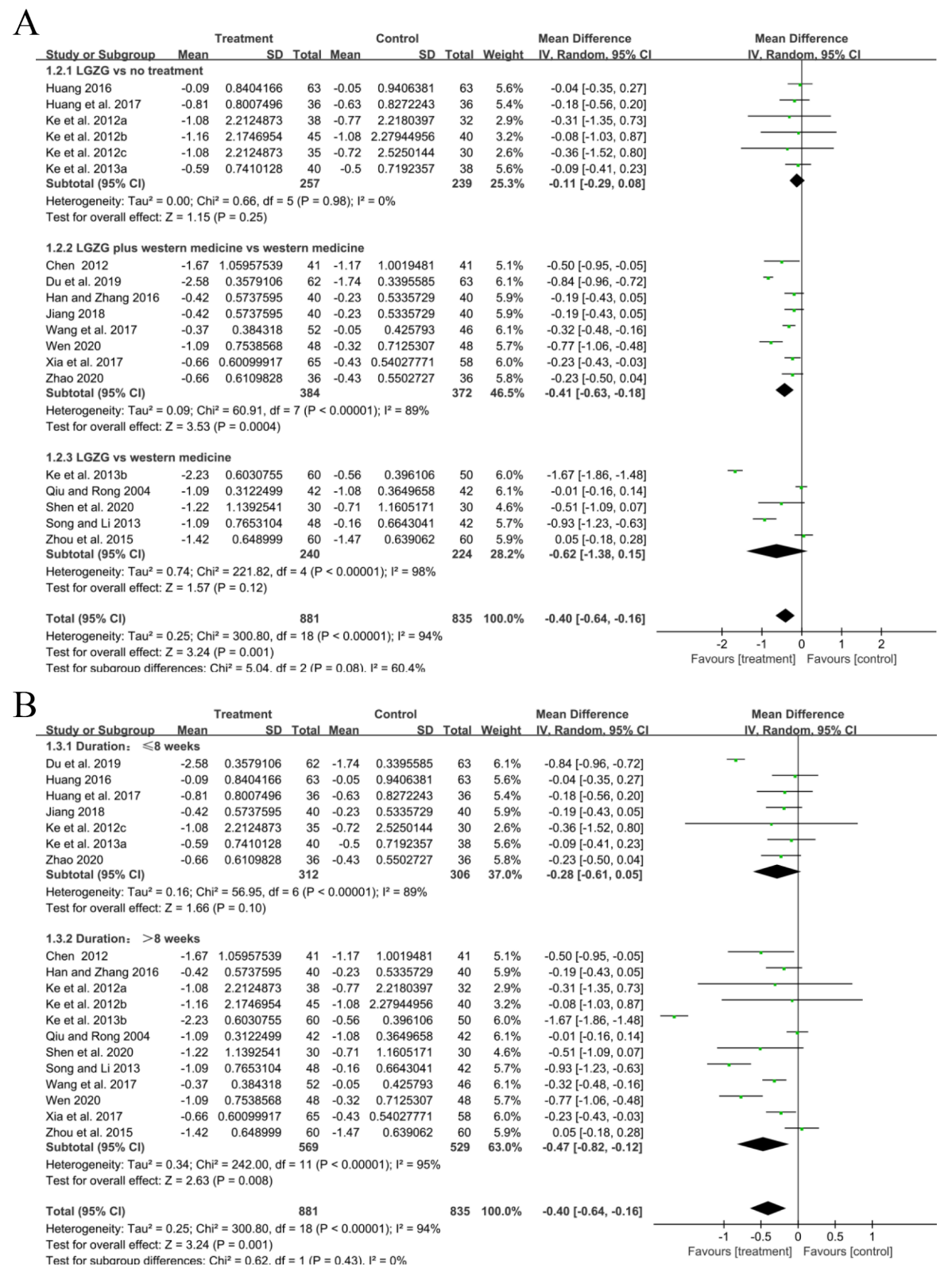


**Figure S2**. Subgroup analyses for TG according to types of intervention and control (A), and duration of intervention (B).


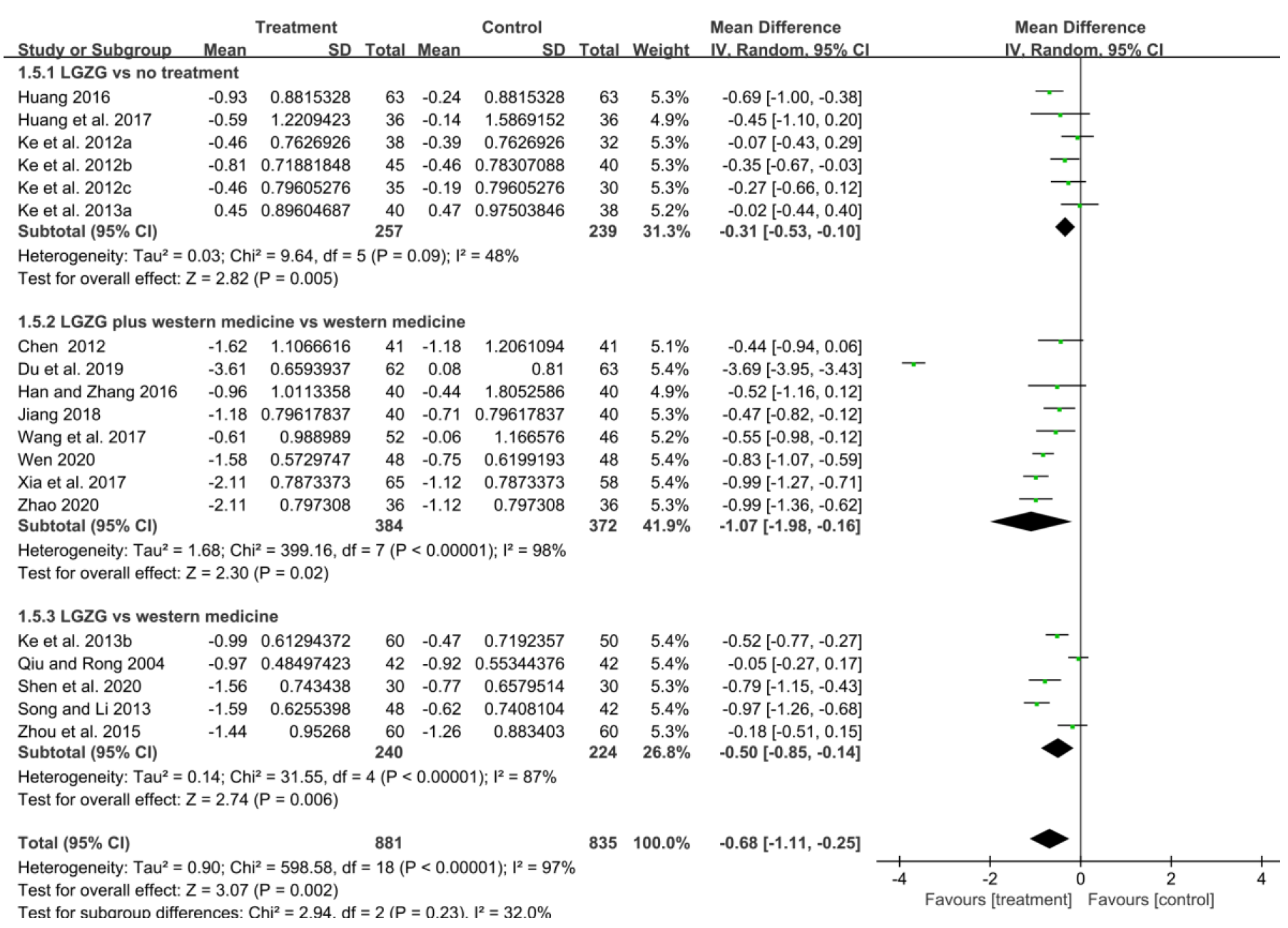


**Figure S3**. Subgroup analyses for TC according to types of intervention and control.


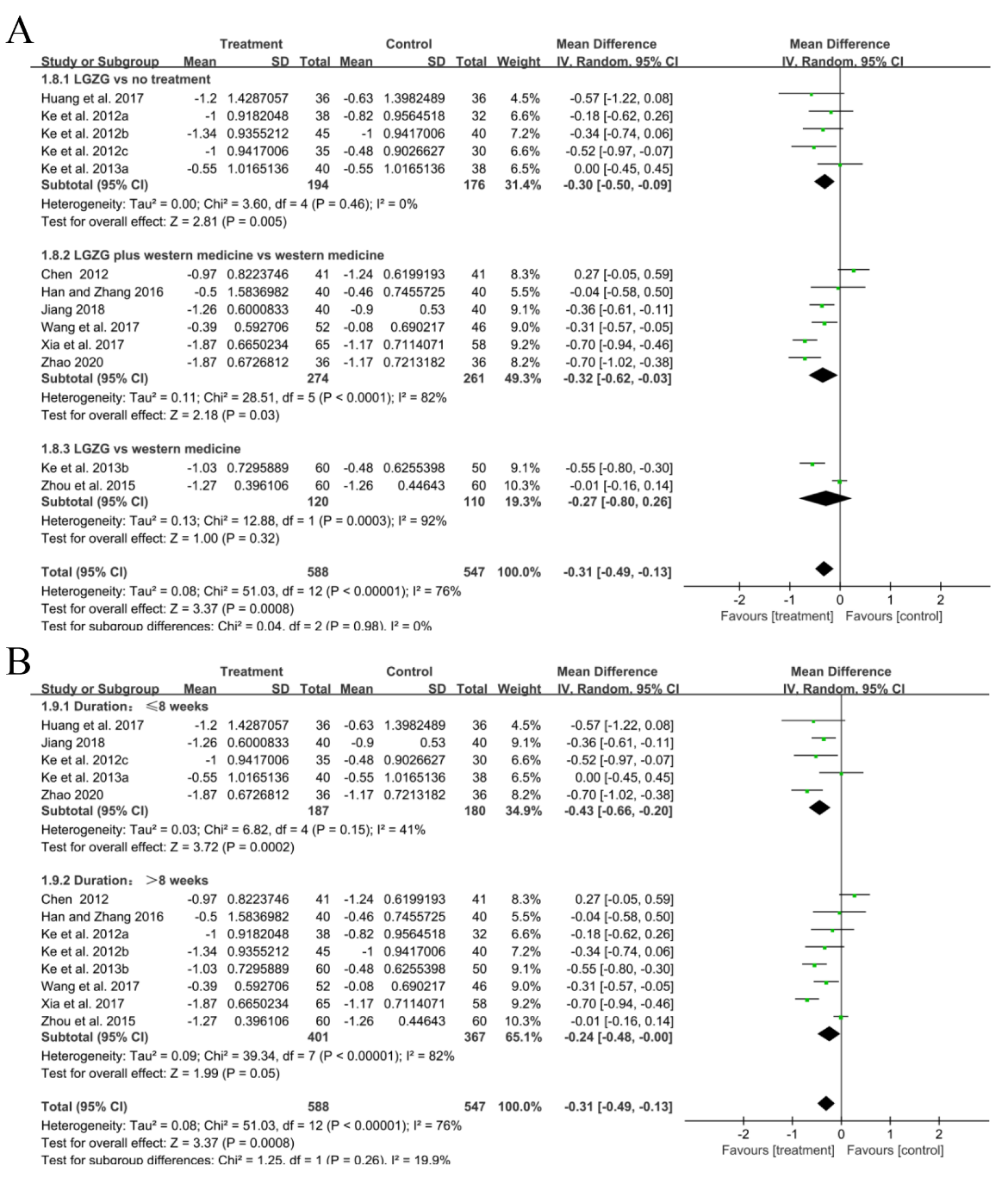


**Figure S4**. Subgroup analyses for LDL-c according to types of intervention and control (A), and duration of intervention (B).


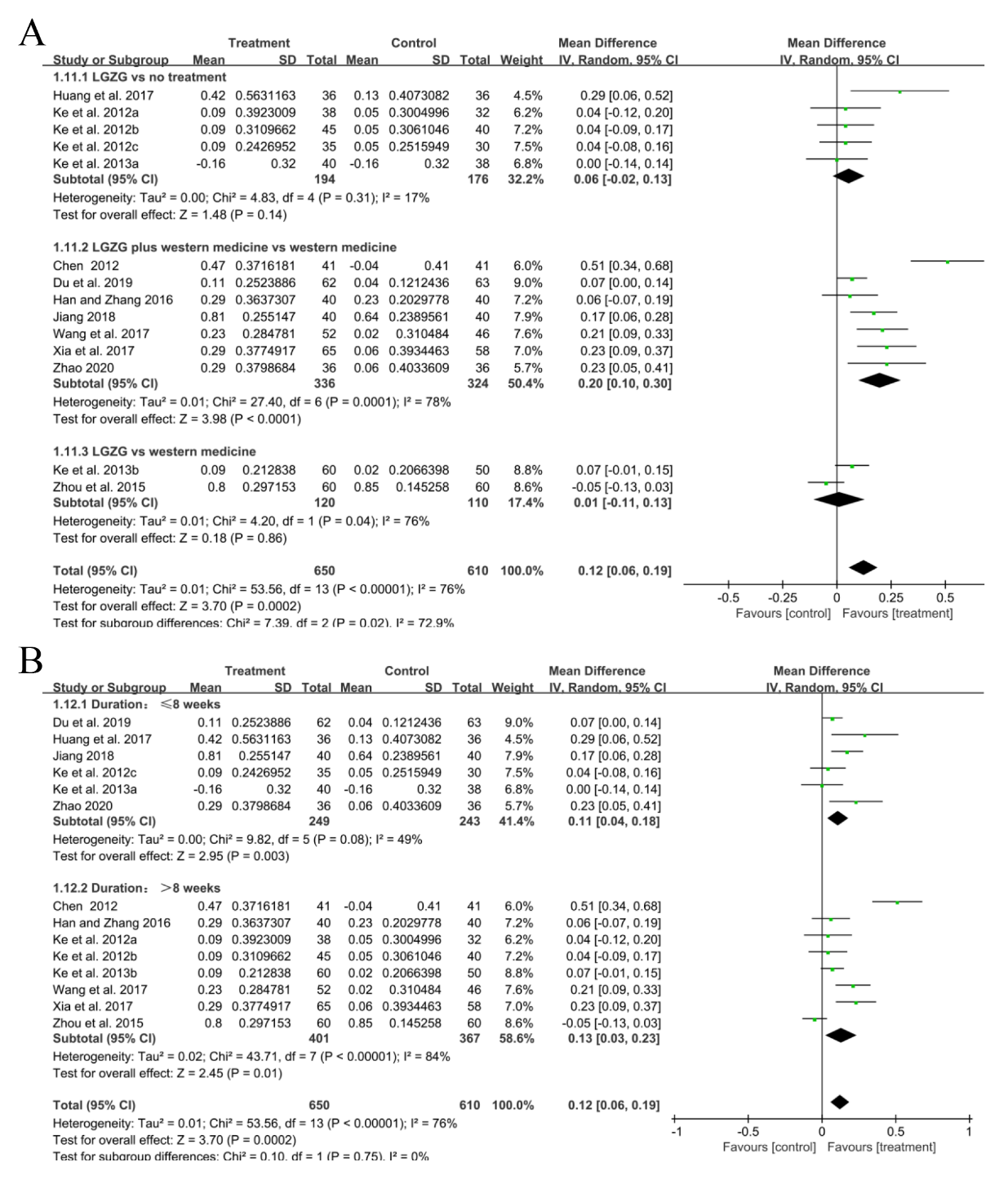


**Figure S5**. Subgroup analyses for HDL-c according to types of intervention and control (A), and duration of intervention (B).


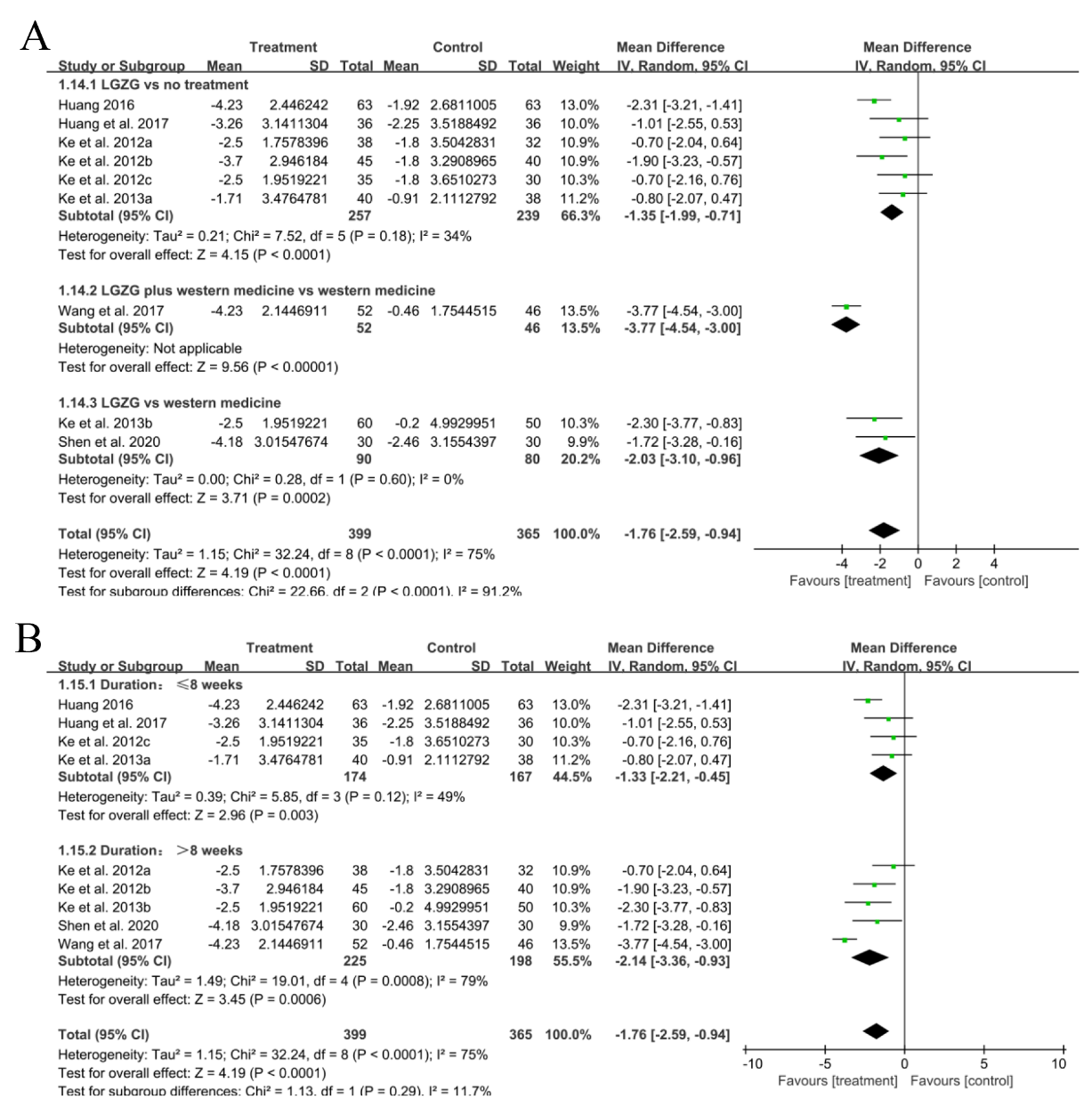


**Figure S6**. Subgroup analyses for BMI according to types of intervention and control (A), and duration of intervention (B).


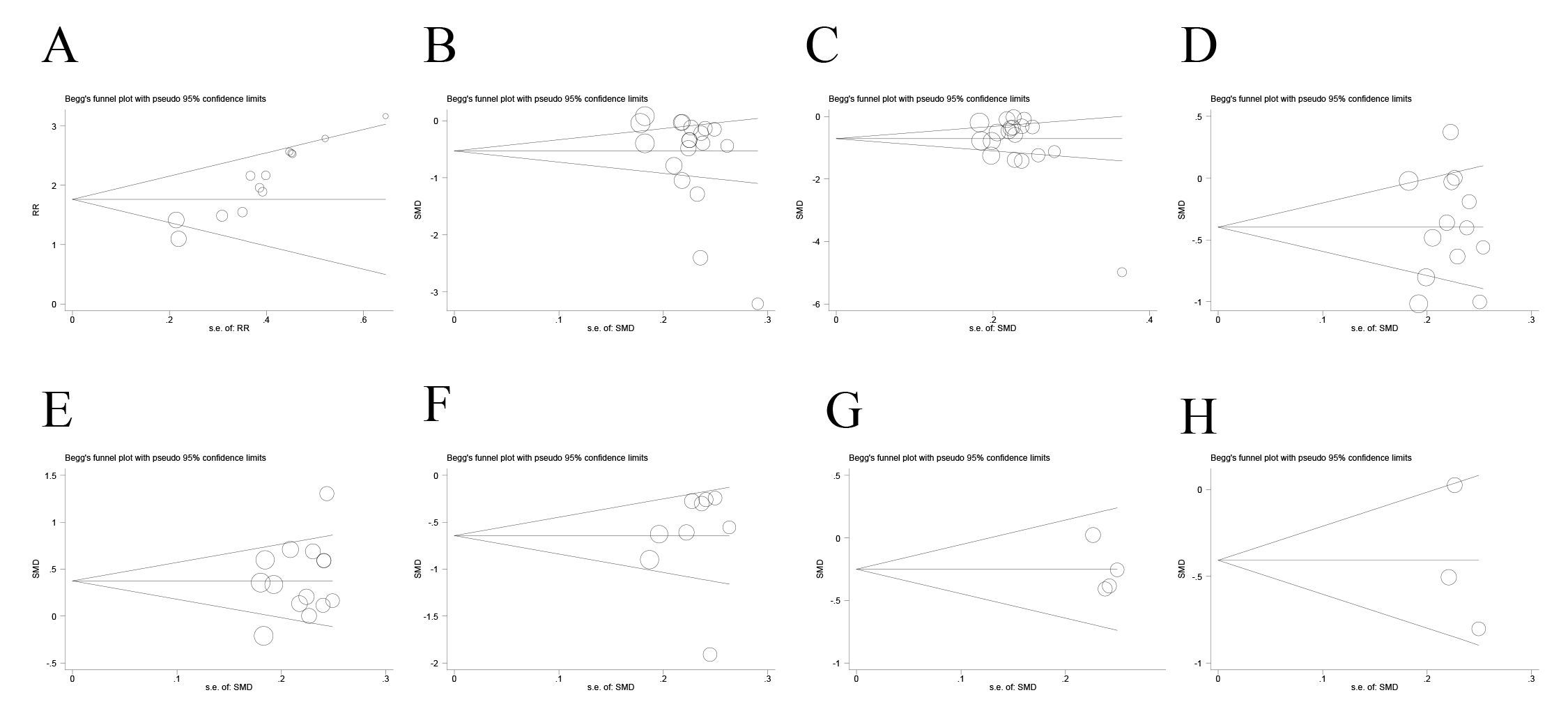


**Figure S7**. Bgger's regression analyses for publication bias. (A) Effective rate; (B) TG; (C) TC; (D) LDL-c; (E) HDL-c; (F) BMI; (G) BW; (H) WC. “O” is a size graph symbol for weights of every included study.


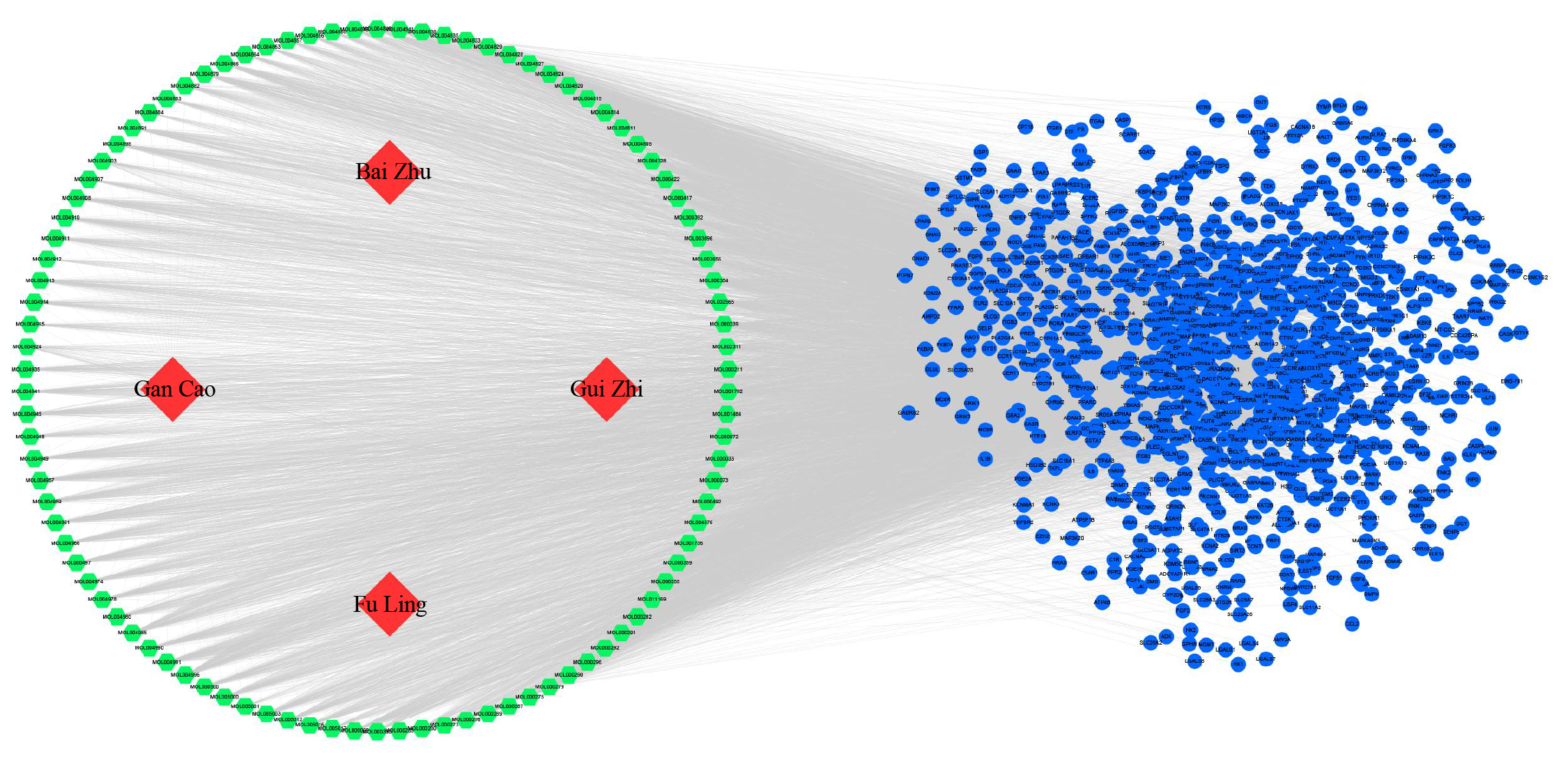


**Figure S8**. Network plot of the active compounds of LGZG and related targets. The red diamonds, green hexagons and blue circles represent the four herbs of LGZG, active components of LGZG and relevant protein targets, respectively.

**References**

1. Yao L, Wei J, Shi S, et al. "Modified lingguizhugan decoction incorporated with dietary restriction and exercise ameliorates hyperglycemia, hyperlipidemia and hypertension in a rat model of the metabolic syndrome," *BMC Complementary and Alternative Medicine, vol.* 17, no. 1, p. 132, 2017.

2. Yang L, Lin W, Nugent CA, et al. "Lingguizhugan Decoction Protects against High-Fat-Diet-Induced Nonalcoholic Fatty Liver Disease by Alleviating Oxidative Stress and Activating Cholesterol Secretion," *International Journal of Genomics, vol.* 2017, p. 2790864, 2017.

3. Liu T, Yang LL, Zou L, et al. "Chinese medicine formula Lingguizhugan decoction improves Beta-oxidation and metabolism of Fatty Acid in high-fat-diet-induced rat model of Fatty liver disease," *BMC Complementary and Alternative Medicine, vol.* 2013, p. 429738, 2013.
